# Engineering chloroplast development in rice through cell‐specific control of endogenous genetic circuits

**DOI:** 10.1111/pbi.13660

**Published:** 2021-08-18

**Authors:** Dong‐Yeon Lee, Lei Hua, Roxana Khoshravesh, Rita Giuliani, Indrajit Kumar, Asaph Cousins, Tammy L. Sage, Julian M. Hibberd, Thomas P. Brutnell

**Affiliations:** ^1^ Donald Danforth Plant Science Center St. Louis MO USA; ^2^ Department of Plant Sciences University of Cambridge Cambridge UK; ^3^ Department of Ecology and Evolutionary Biology the University of Toronto Toronto ON Canada; ^4^ Department of Biology the University of New Mexico Albuquerque NM USA; ^5^ School of Biological Sciences Washington State University Pullman WA USA; ^6^ Biotechnology Research Institute Chinese Academy of Agricultural Sciences Beijing China; ^7^ Joint Laboratory for Photosynthesis Enhancement and C_4_ Rice Development Biotechnology Research Institute Chinese Academy of Agricultural Sciences Beijing China

**Keywords:** C_4_ photosynthesis, dCas9‐mediated transcriptional activation, rice bundle sheath, chloroplast development

## Abstract

The engineering of C_4_ photosynthetic activity into the C_3_ plant rice has the potential to nearly double rice yields. To engineer a two‐cell photosynthetic system in rice, the rice bundle sheath (BS) must be rewired to enhance photosynthetic capacity. Here, we show that BS chloroplast biogenesis is enhanced when the transcriptional activator, *Oryza sativa Cytokinin GATA transcription factor 1* (*OsCGA1)*, is driven by a vascular specific promoter. Ectopic expression of *OsCGA1* resulted in increased BS chloroplast planar area and increased expression of photosynthesis‐associated nuclear genes (PhANG), required for the biogenesis of photosynthetically active chloroplasts in BS cells of rice. A further refinement using a DNAse dead Cas9 (dCas9) activation module driven by the same cell‐type specific promoter, directed enhanced chloroplast development of the BS cells when gRNA sequences were delivered by the dCas9 module to the promoter of the endogenous *OsCGA1* gene. Single gRNA expression was sufficient to mediate the transactivation of both the endogenous gene and a transgenic GUS reporter fused with *OsCGA1* promoter. Our results illustrate the potential for tissue‐specific dCas9‐activation and the co‐regulation of genes needed for multistep engineering of C_4_ rice.

## Introduction

Increasing demands placed on the global food supply indicate that a 50%–70% increase in agricultural output will be necessary by 2050 (Jaggard *et al*., 2010). Yet yields in rice, a primary staple consumed by over half of the world’s population, have remained relatively flat over the past decade, suggesting that yield gains driven by “green revolution” engineering have been maximized under our current production systems (Grassini *et al*., 2013). The installation of a C_4_ photosynthetic system into rice has the theoretical potential to double current rice yields (von Caemmerer *et al*., 2012; Hibberd *et al*., 2008) through improved photosynthetic efficiencies while reducing water and nitrogen demands. However, both fundamental discoveries and several technological hurdles must be overcome to achieve this goal (Sage and Zhu, 2011). As a central feature to most C_4_ systems, photosynthetic activities are partitioned between two cell types, the BS and the mesophyll (M) (Hibberd and Covshoff, 2010). The chloroplasts of the BS must decarboxylate malate and perform selective Calvin Cycle activities with reduced PSII activities, whereas the M cell plastids perform linear electron transport and reduce oxaloacetate into malate (Hatch, 1987). Unlike the BS plastids in a C_3_ plant, in a C_4_ context, the BS plastids serve an essential role in photosynthesis and therefore the number and or size of the BS plastids in rice must be increased to support the C_4_ cycle. Thus, in order to install a C_4_ system into rice, cell‐type specific promoters will be required to drive differential gene expression. It has been estimated that at least 20 genes will need to be differentially expressed (DE) between BS and M to install a minimal C_4_ system (Peterhansel, 2011). Another challenge is our limited understanding of the regulatory networks that drive both anatomical features and biochemical pathways underlying C_4_ photosynthesis. *In lieu* of these transcriptional regulators, single genes must be manipulated with the goal of targeting specific biochemical activities that may be insufficient to drive an operational C_4_ cycle.

Here, we have utilized a transcriptional activator that promotes chloroplast development through enhanced cytokinin responsiveness (Hudson *et al*., 2013; Naito *et al*., 2007; Reyes *et al*., 2004) to manipulate chloroplast architecture specifically in the BS of rice. This was achieved by driving *OsCGA1* expression with the use of the *glycine decarboxylase p‐subunit promoter* (*pFtGLDp*), originally characterized in *Flaveria trinervia (*Engelmann *et al*., 2008 *)*. Ectopic expression of Os*CGA1* resulted in increased accumulation of photosynthesis‐associated nuclear gene (PhANGs) transcripts including peripheral proteins for the plastid encoded RNA polymerase (PEP) complex, peripheral proteins of the plastid ribosome and light harvesting complexes in the BS. Histological characterizations and confocal imaging also revealed enhanced photosynthetic development in the BS cells of the transgenic plants. Importantly, the use of a two component dCas9/gRNA system to deliver a transcriptional activation module to the promoter of an endogenous rice gene opens the possibility of co‐regulating suites of genes in a cell‐type specific manner in rice and addresses a fundamental challenge in engineering C_4_ photosynthesis or similarly complex systems.

## Results

Previous characterizations of *Cytokinin GATA transcription factor 1* (*CGA1*) misexpression in *O.sativa var*. Nipponbare and *Arabidopsis thaliana* suggested that *CGA1* functions cell autonomously to drive the expression of genes associated with chloroplast differentiation (Chiang *et al*., 2012; Hudson *et al*., 2011,2013). To investigate the phenotypic consequences of CGA1 overexpression in *O.sativa* var. Kitaake, a short‐statured and largely photoperiod insensitive rice variety, *OsCGA1* was introduced into rice calli under the control of the *maize ubiquitin 1* promoter (Christensen and Quail, 1996). Ectopic expression of *OsCGA1* resulted in photosynthetic differentiation of callus tissue and partial shoot regeneration even when cytokinins were excluded from the growth media (Figure S1). Due to the pleiotropic growth defects associated with constitutive *OsCGA1* expression on regeneration and growth, we opted for cell‐specific expression of *OsCGA1* using the relatively weak *FtGLDp* promoter to enhance *OsCGA1* expression in vascular tissues including the bundle sheath (Engelmann *et al*., 2008). In rice, *pFtGLDp::GUS* fusions conditioned a weak vascular profile of expression that was dependent on the absence of intronic sequences in the β‐glucuronidase reporter (Figure S2, Table S2). Interestingly, when intron sequences were inserted into the GUS reporter, it conditioned uniform leaf expression (Figure S2).

**Figure 1:**
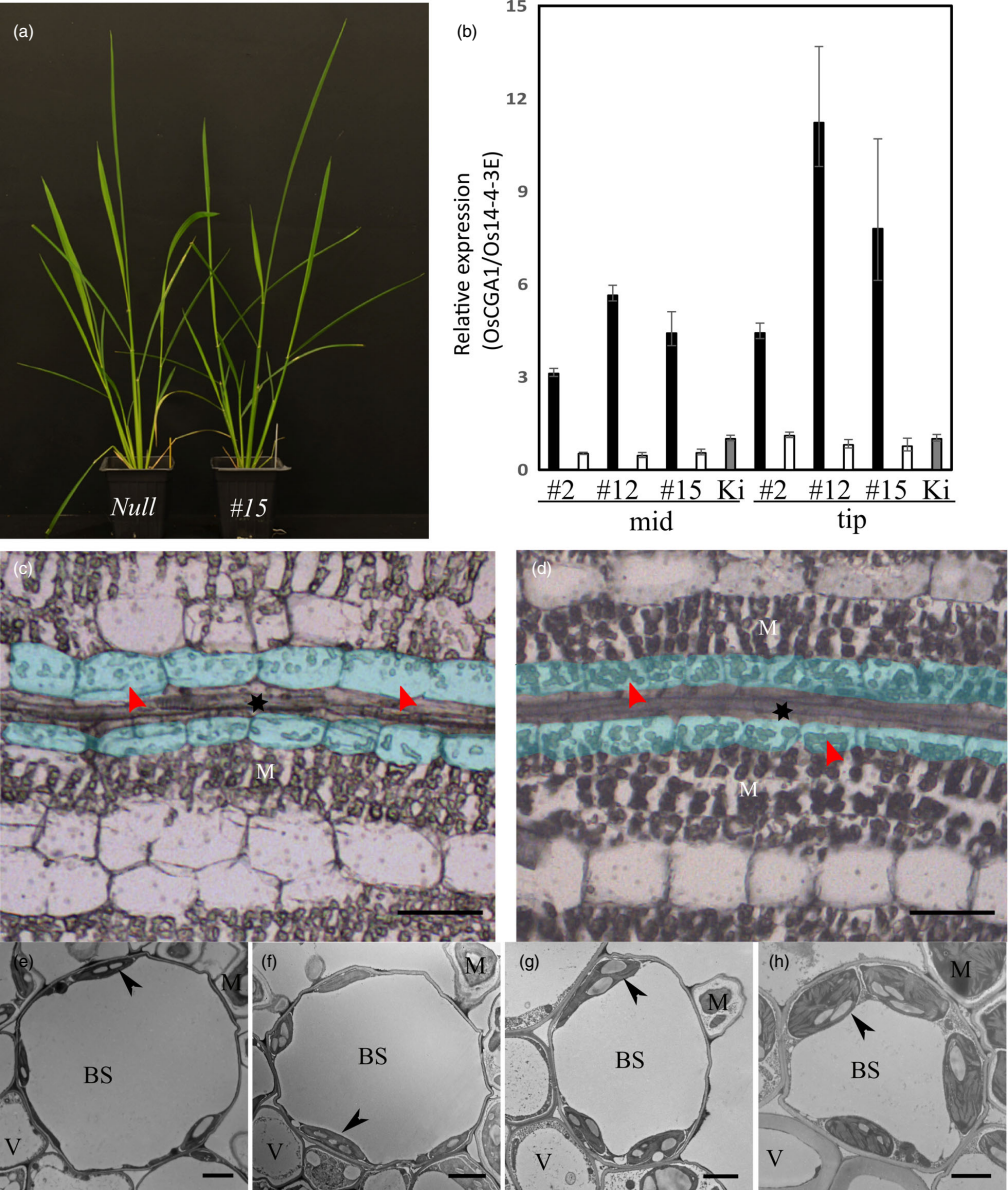
*pFtGLDp::OsCGA1* plant appearance, transgene expression and BS chloroplast phenotypes. (a) *pFtGLDp::OsCGA1* carrying plant (right) shows a pronounced green sheath compared to the null segregant (left). (b) Expression level of *pFtGLDp::OsCGA1* in 3 independent transgenic events (#2, #12 and #15). Black bars and empty bars indicate segregating homozygotes and nulls, respectively. Kitaake WT is shown as a grey bar and 2 cm leaf segments from two individuals were pooled for each sample. The bars represent mean +SD and −SEM of three biological replicates. Mid, middle region of leaf; Tip, Tip region of leaf. (c and d) BS chloroplast phenotype of *FtGLDp::OsCGA1* transgenic plant. Paradermal sections of leaf tissue were imaged using light microscopy prior to sampling for laser capture microdissection; (d) *pFtGLDp::OsCGA1* #12 homozygote and (c) null segregant. The BS cells are shaded in light blue, M denotes mesophyll cells and * denotes vascular tissue. Arrowheads indicate the chloroplasts in BS. Bars, 5 μm. (e–h) Transmission electron micrographs of fourth leaves of (e) null segregant and transgenic lines (f) #2–13, (g) #12–15 and (H) #15–19. Arrows denote the chloroplasts. BS, bundle sheath; M, mesophyll; V, vascular tissue. Bars, 2 μm.

**Figure 2:**
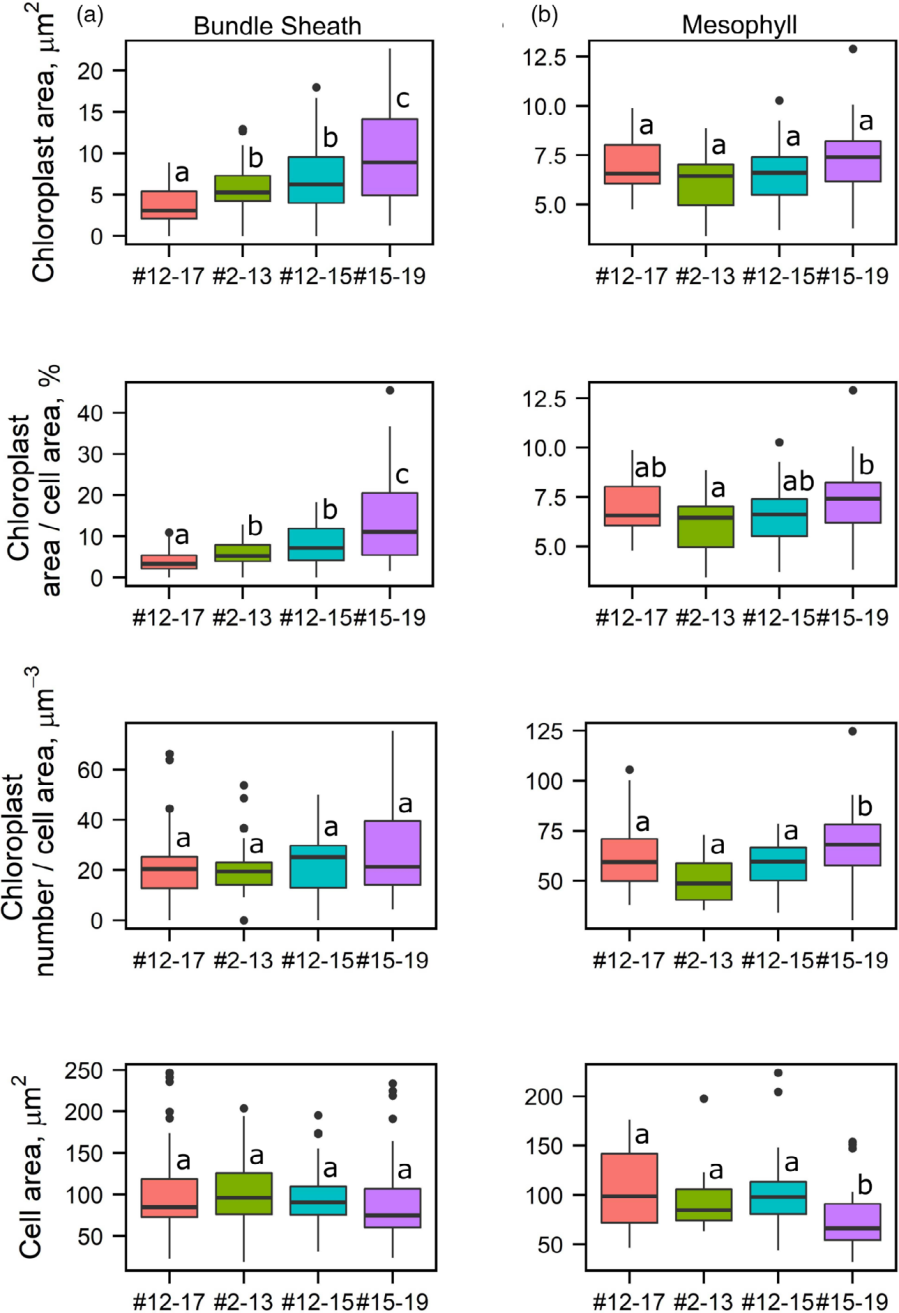
Quantification of chloroplast phenotypes in *pFtGLDp::OsCGA1* transgenic plants. Quantification of chloroplast area and number in the bundle sheath (a) and mesophyll (b) cells from TEM images of WT (#12–17) and three independent transgenic events (#2–13, #12–15 and #15–19). Within each box, horizontal black lines denote median values. Top and bottom of boxes indicate the 25th and the 75th percentile of each group’s distribution of values. Vertical extending lines and dots denote adjacent values and observations outside the range, respectively. Values (^a–d^) with the same letters represent no significant difference (*P* > 0.05) when a Kruskal–Wallis one‐way analysis of variance was performed followed by a Dunn's *post hoc* test using an R script.

When the *FtGLDP* promoter was used to drive *OsCGA1*, calli were regenerated using standard tissue culture procedures and resulted in plants with general growth characteristics and phenotypes that were not easily distinguishable from the null segregant lines until after flowering when we noticed transgenic plants were slightly greener than null lines (Figure 1). Among 22 independent transgenic events, three single copy insertions conditioned a 4 ~ 13 fold increase in *OsCGA1* expression as determined by RT‐PCR (Figure 1b). Siblings of three single copy transgenic events (#2, #12, #15) and one partial tandem duplication event (#4) were characterised at T1 and T2 generations (Table S2). Histological characterizations of transgenic lines revealed that chloroplast size in the BS was enhanced (Figure 1). Quantification of the plastid area using EM analysis indicated that the BS plastids of transgenics had increased up to 3.5 fold in size relative to the chloroplasts of nulls (Figure 2a). In two of the transgenic lines, the M chloroplast area was not significantly different in transgenics relative to nulls. However, in one line mesophyll cell size was reduced (# 15–19, Figure 2b). As this phenotype was observed in only one event it is not clear if ectopic expression of OsCGA1 may also mediate changes in M cell architecture (Figure 2b). Ultrastructural characterizations indicated that granal stacking and the size of starch granules were not significantly different from null lines. Thus, the increased size of the plastids did not obviously affect internal features of the plastids (Figure 1 and Figure S3). In addition to larger chloroplasts, mitochondria number and area were also increased in the BS of transgenic lines (Table S1), potentially indicative of enhanced photorespiratory activities in the BS plastids. Immunodetection of *RUBISCO*, *RUBISCO activase*, *fructose 1,6‐bisphosphatase (FBPase)* and the mitochondrial enzyme *glycine decarboxylase (GLD)* indicated these enzymes are present in the BS chloroplasts and mitochondria of transgenic and null lines suggesting that BS cells could support photosynthetic activities in both WT and transgenic lines (Figure S3). To determine if the increase in BS chloroplast area could enhance photosynthetic performance, leaf net CO_2_ assimilation rates (*A*) versus intercellular CO_2_ partial pressure (Ci) was measured in two independent *pFtGLDp::OsCGA1* events and corresponding nulls under atmospheric O_2_ partial pressure of 18.4 kPa. Additionally, Rubisco activity, chlorophyll *a* + *b* content, chlorophyll *a*/*b* ratio, specific leaf mass (SLM) and total nitrogen were also measured. However, as shown in Figure S4, there were no significant differences between the transgenics and WT plants for any of these parameters. These results suggest that despite an increase in BS plastid area and possible enrichment of some photosynthetic enzymes, these enhancements were insufficient to impact global photosynthetic performance of the plants. This is perhaps not surprising given the limited access of CO_2_ to the BS chloroplasts that are embedded within leaf tissue and surrounded by multiple dense layers of photosynthetically active M cells. Nevertheless, these results suggest that manipulation of *OsCGA1* could provide an avenue to enhance BS photosynthesis if a mechanism to deliver CO_2_ to the BS was also installed.

To further investigate the molecular consequences by *OsCGA1* misexpression, we performed RNA‐seq analysis on four developmentally distinct leaf segments, base, −1 (a region covered by the outer leaf), +4 (middle region of emerged leaf) and tip from the emerging fourth leaves, similar to the leaf gradient scheme in (Li *et al*., 2010). As shown in Figure S5, these data suggest that given the low vascular activity of the *FtGLDp* promoter and the low BS to M cell ratio in leaf segments, we lacked the resolution to monitor global changes in gene expression using whole tissue segments.

**Figure 3:**
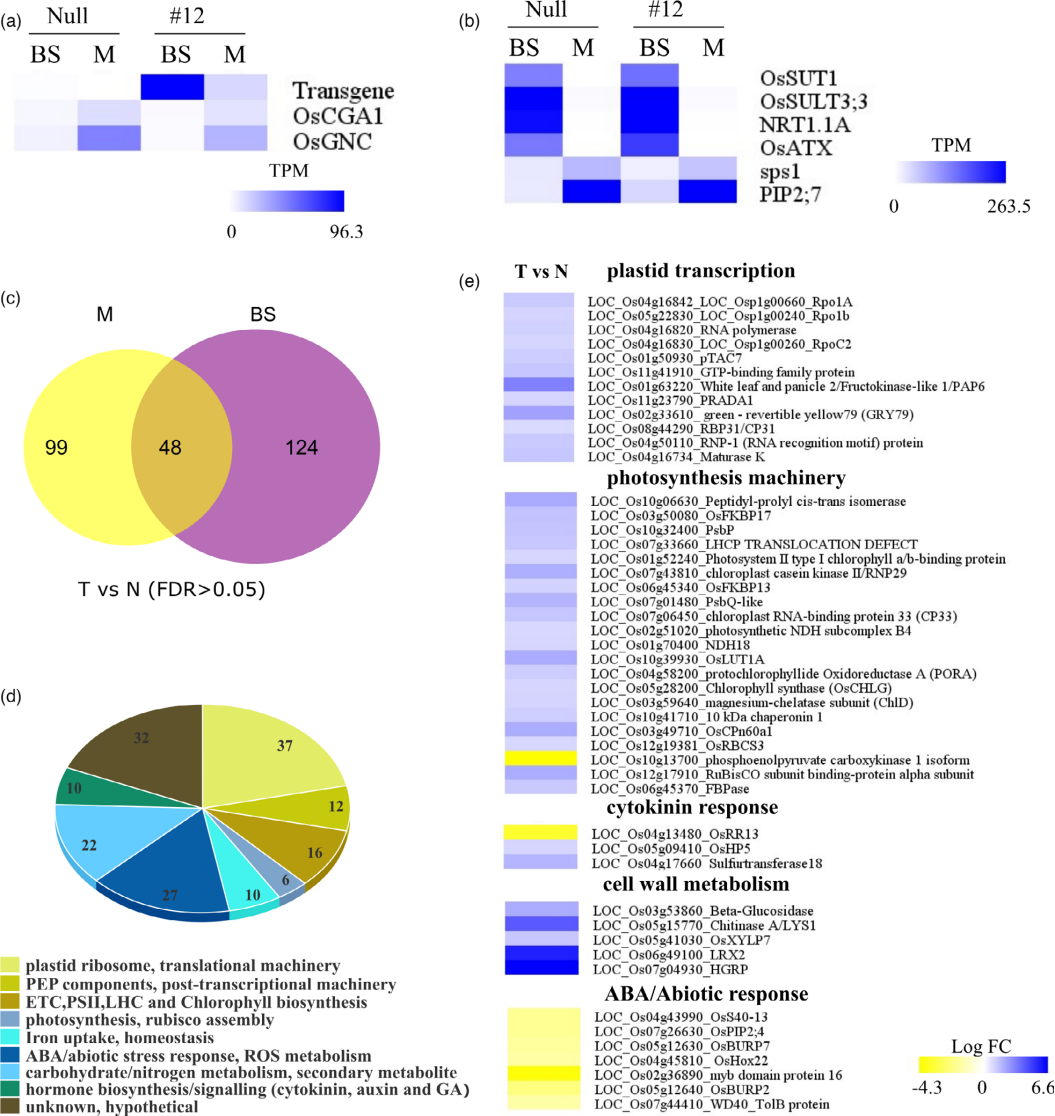
Expression of transgene and marker genes in LCM‐RNAseq and DE gene analysis in the BS of *pFtGLDp::OsCGA1* plants. (a) Expression of endogenous *OsCGA1*, *OsGNC* and transgenic *OsCGA1* in BS and M from LCM RNAseq. Transgenic *OsCGA1* and endogenous *OsCGA1* transcripts are discriminated by read mapping to unique regions in the 3’ end of transcripts (*OsCGA1‐Nos* terminator fusion transcripts and native *OsCGA1* transcripts). Mean Transcripts Per Million (TPM) from three biological replicates of BS and M are represented as a color‐scale in the heat map. Data for the *pFtGLDp::OsCGA1* #12 (#12) and the null are shown. (b) Expression patterns of vascular bundle and M markers in BS and M LCM RNAseq. Vascular bundle marker genes: *OsSUT1* (LOC_Os03g07480), *OsSULT3;3* (LOC_Os04g55800), *NRT1.1A* (LOC_Os08g05910), *OsATX* (LOC_Os01g67010); mesophyll marker genes: *sps1* (LOC_Os01g69030), *PIP2;7* (LOC_Os09g36930). (c) Summary of DE genes in BS strands and M strands between transgenics and nulls. There were 48 genes expressed in both BS and M but differentially expressed between transgenic and null plants. (d) Manually curated gene categorizations of DE genes from BS. Number of genes in each category are shown. (e) Heatmap of subsets of DE genes enriched in the gene clusters including plastid transcription, photosynthesis machinery, cytokinin response, cell wall metabolism and ABA/Abiotic responses. T vs. N, Transgenic versus Null.

To enhance the sensitivity of detecting DE genes, we performed Laser Capture Microdissection (LCM) on the mid‐region of leaves from T3 transgenic lines and their null segregants of strongest #12 event (Table S2). Cross‐sections of Steedman’s wax embedded leaf tissue were used to harvest bundle sheath strands (BSS) and Mesophyll (M) for RNA‐Seq analysis (Figure S6). An increase in BS chloroplast size was notable in transgenic lines under the LCM microscope (Figure 1c ~ d). LCM captured BS and M strips displayed the pronounced differential expression of vascular marker and mesophyll marker gene expression indicating little cross‐contamination (Figure 3b). As expected, endogenous *OsCGA1* was expressed at higher levels in M cells relative to BS in both transgenic and null lines. However, *CGA1* expression driven from the transgene was 6.2 times higher in BS than in M in transgenic lines, corroborating the plastid phenotypes associated with *FtGLDp* promoter activity in rice (Figure 3a). Differential expression analysis between transgenic and null lines led to the identification of 172 DE and 147 DE genes in BS and M strips, respectively, with an FDR cut off 0.05 (Figure 3c). Pageman analysis (Usadel *et al*., 2006) showed that photosystem II, electron transport chain, vitamin biosynthesis and plastid ribosomal machinery genes are enriched in transgenic BS transcriptome relative to the null counterpart (Figure S7). In contrast, immune response, protein degradation and vacuolar ATPase activity are markedly attenuated in the transgenic BS **(**Figure S7). Surprisingly, a total of 41.2% of DEG are proportioned to a broad range of photosynthesis‐associated genes (PhANGs), including nuclear‐encoded plastid ribosomal/translational machinery genes (21.5%), peripheral proteins for PEP complex (6.9%), chlorophyll biosynthesis, components of light harvesting complex and photosynthetic enzymes (FBPase and SSU) (Figure 3d). Interestingly, nucleus‐encoded PEP peripheral subunits, *pTAC7*, *PAP6/FLN1,* are up‐regulated together with the plastome‐located core units, *rpoA*, *rpoB* and *rpoC2* (Figure 3e) in transgenic BS (He *et al*., 2018; Steiner *et al*., 2011). PEP complex activity is largely dependent on nucleus encoded PEP‐associated proteins (PAP) and is responsible for the photosynthetic gene expression in chloroplast development (Börner *et al*., 2015; Toyoshima *et al*., 2005). In contrast, nucleus encoded RNA polymerase (NEP), responsible for housekeeping gene expression in plastids (Demarsy *et al*., 2006; Hajdukiewicz *et al*., 1997), was not altered in *OsCGA1* BS (data not shown). Other plastid transcription‐associated genes, including Os11g41910 (GTP binding protein) (Ye *et al*., 2017), Os11g23790 (homologous to *AtPEP‐RELATED DEVELOPMENT ARRESTED 1*) (Qiao *et al*., 2013), plastid post transcriptional machinery genes, nucleus encoded plastid translation machinery, electron transport chain components, light harvesting complexes, and chlorophyll biosynthesis pathway genes also accumulated to higher levels in the BS of transgenic plants (Figure 3e, Table S5). Collectively, these data suggest that ectopic expression of CGA1 is sufficient to mediate a transcriptional cascade leading to a global increase in photosynthetic machinery. Supporting this trend, we observed a significant enrichment in chloroplast biogenesis pathways genes with a total of 46.5%% of DEG with at least one predicted plastid localization signal (Sperschneider *et al*., 2017) (Table S5).

In addition to photosynthesis‐associated genes, several cytokinin response and cell wall metabolism genes accumulated to higher levels in the transgenics. The exception was *OsRR13*, a potential inhibitor of cytokinin signal transduction based on its *arabidopsis* homolog *ARR22* (Figure 3E) (Du *et al*., 2007; Wallmeroth *et al*., 2017,2019). This suggests that cytokinin response may be enhanced in BS cells ectopically expressing *OsCGA1*. Furthermore, a subset of ABA‐ and drought‐inducible genes are markedly decreased in BS of transgenics (Figure 3e, Table S5). As ABA is generally associated with the degradation of chloroplasts and increased senescence (Zhuang and Jiang, 2019), these data are consistent with an enhanced chloroplast biogenesis program in the BS of transgenics.

**Figure 4:**
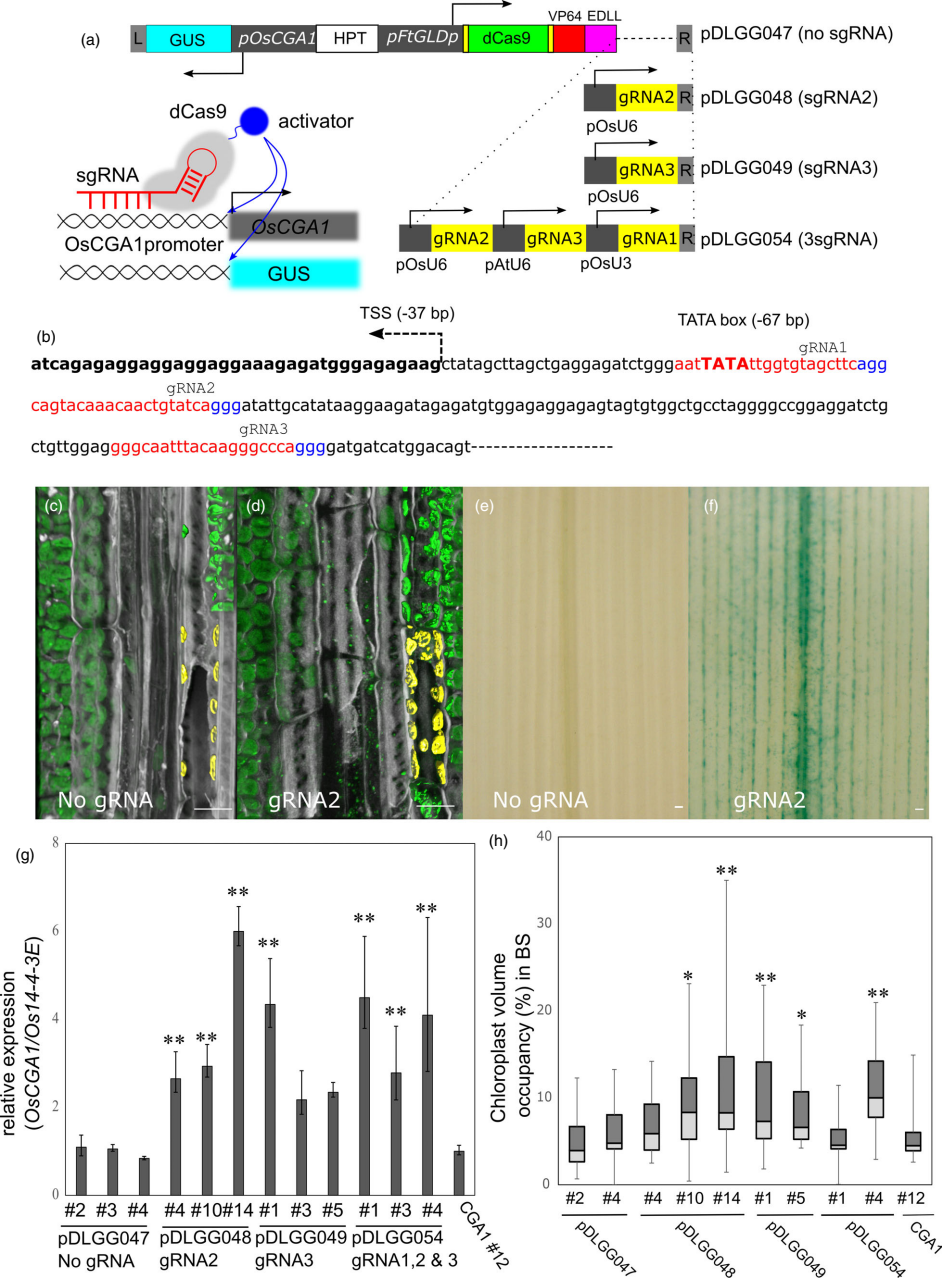
Tissue specific activation of *OsCGA1* by dCas9‐AD constructs. (a) Schematic of dCas9‐mediated transcriptional activation. All dCas9 activation constructs have similar structures with the exception of gRNA sequences and number of gRNA expression modules to recruit dCas9‐ADs to the upstream region of both the endogenous *OsCGA1* gene and *OsCGA1promoter‐GUS* reporters. The 2.1 kb upstream region of *OsCGA1* was fused to the GUS gene as a proxy to monitor the tissue specificity of gene activation conferred by the *FtGLDp* promoter driven *dCas9‐VP64‐EDLL*. The pDLGG047 construct contains only the dCas9‐VP64‐EDLL cassette without a gRNA expression module and pDLGG048, pDLGG049 have a single gRNA module driven by the *OsU6snRNApolymerase* promoter. pDLGG054 contains three gRNAs expression modules driven by different *snRNApolymerase* promoters (detailed in Table S3). Left inset figure showing the schematic of dCas9*‐*mediated activation for both the endogenous *OsCGA1* gene and the *pOsCGA1::GUS* reporters. HPT, hygromycin resistance cassette. Arrows indicate the direction of transcription. (b) gRNAs designed to the proximal region of the transcriptional start site of *OsCGA1*. gRNA binding regions and PAM sites are highlighted as red and blue, respectively. TSS, transcriptional start site. (c and d) 3D‐reconstructed confocal images of longitudinal leaf section in the BS of dCas9 activation lines, (c) dCas9‐ADs only transgenic (pDLGG047 #4) and (d) transgenic with gRNA2 (pDLGG048 #1). Chlorophyll autofluorescence and cell wall staining by calcofluor‐white are pseudo‐coloured as green and grey, respectively. Chloroplasts within a BS cell are rendered for volume quantification and highlighted as yellow pseudo colour. Scale bar, 10 μm. (e–f) GUS reporter activation by dCas9‐mediated transactivation, (e) dCas9‐ADs only transgenic (pDLGG047 #4) and (f) transgenic with gRNA2 (pDLGG48 #14). Scale bar, 10 μm. (g) Transcript accumulation difference of *OsCGA1* by gRNA position. 2 cm long‐mid regions from the emerging (5th) leaf were harvested for RT‐qPCR. Three independent events from each construct are assessed for *OsCGA1* expression. Each bar refers to the mean +SD and −SEM of indicated events (2 ~ 4 T1 siblings, three PCR replicates). The letters above the bars note significant differences (**P < 0.01 as analysed using one‐way ANOVA followed by a Tukey’s *post hoc* test) compared to the expression level of pDLGG047 (no gRNA) control lines. (h) Box plot of chloroplast volume occupancy in the BS of various *OsCGA1‐dCas9* activation events. The letters above the bars denote significant differences (**P < 0.01, *P < 0.05 as analysed using one‐way ANOVA followed by a Tukey’s *post hoc* test) compared to the chloroplast volumes of pDLGG047 (no gRNA) control lines.

To exploit the *FtGLDp* promoter in driving multigene vascular specific expression, we employed the dCas9 activation strategy to up‐regulate the endogenous *OsCGA1* in a tissue‐specific manner (Gilbert *et al*., 2013; Lowder *et al*., 2018). To demonstrate our proof‐of‐concept, we constructed a series of multigene cassettes to assess the transactivation capacity using a series of gRNAs directing dCas9‐activation modules to specific regions of the *OsCGA1* promoter (Figure 4). To confer tissue specificity, we utilized the *FtGLDp* promoter to drive a *dCas9* gene fused with *VP64* and *EDLL*, two tandem activation domains (ADs) (Figure 4a). A 2.1 kb upstream sequence of the *OsCGA1* gene was used to drive GUS reporter expression to validate tissue specific transactivation (Figure 4a). Multiple gRNAs were designed to target a region surrounding the TATA box of the *OsCGA1* promoter and driven with the constitutive *U6/U3 snRNApol promoter* (Figure 4b). Prior to the generation of stable transgenics, we tested the positional effects of gRNAs on *pOsCGA1::GUS* gene activation using a constitutively expressed *pZmUbi1::dCas9‐ADs* construct in heterologous tobacco leaf assays (Figure S8). Among five gRNAs tested, gRNA2 and gRNA3 were the most effective in the activation of the *pOsCGA1::GUS* reporter (Figure S8) and resulted in mature plant phenotypes similar to *pFtGLDp::OsCGA1*. Although we introduced more constructs in rice, poor regeneration, low fertility and occasional loss of T‐DNA fragments were prevalent throughout the primary transgenic and subsequent generations (data not shown). Therefore, we limited our analysis to four stable constructs, *dCas9‐ADs* only, gRNA2, gRNA3 and multiple gRNA expression modules, in which we obtained more than three independent events (Figure 4). Due to the complexities associated with multigenic transgenes, we evaluated *OsCGA1* transactivation and associated chloroplast phenotypes within single transgenic T1 siblings from low copy transgenic parents (Table S2). Activation levels of endogenous CGA1 varied depending on gRNA positions and transgenic events but showed an approximately 2 ~ 5 fold higher level compared to the expression of the *dCas9‐ADs* only transgenic (Figure 4g). Similar to transient assays, gRNA2 and gRNA3 were effective for endogenous *OsCGA1* activation (Figure 4g). When multiple gRNAs were expressed with different U6/U3 promoters, we did not observe an additive effect on *OsCGA1* expression, indicating a possible steric hindrance or competition between dCas9 proteins for access to the promoter region (Figure 4g). GUS staining of lines carrying *pOsCGA1::GUS* revealed strong staining along the veins (Figure 4f) that was much more distinct than the pattern conditioned by the p*FtGLDp::GUS* construct (Figure S9). Due to the large vacuole of the BS, GUS staining is only apparent in some BS cells when cytosolic portions are captured in 50 μm thick vibratome sections (Figure S9). Consistent with RT‐PCR analysis, gRNA2 and gRNA3 were most effective in driving tissue‐specific GUS reporter activation and also failed to display additive effects with gRNA multiplexing (Figure 4g and Figure S10). In summary, a dCas9 activation module was successfully used to drive transcript accumulation of the endogenous *OsCGA1* and *OsCGA* driven‐GUS expression in a tissue‐specific manner. These results indicate that dCas9‐ADs act in a cell‐autonomous manner and can be utilized as shown here to drive tissue‐specific expression of multiple gene cassettes.

To assess dCas9‐activated BS chloroplast proliferation, we scored the chloroplast volume in BS of transgenic lines using a 3D‐rendering of chlorophyll autofluorescence using confocal laser scanning microscopy on thick longitudinal vibratome sections (Figure 4c ~ d). Chloroplast volume within the BS is highly variable and dependent on position within the vascular bundle. Nevertheless, there was a significant increase in chloroplast volume in the BS of OsCGA1 transactivation lines compared to the control, dCas9‐ADs only cassette (Figure 4h). Two insertion events in particular, pDLGG048 #14 (gRNA2) and pDLGG049 #1 (gRNA3), conditioned a nearly 2.2‐fold increase in average chloroplast volume occupancy relative to the dCas9 only (pDLGG047) event (Figure 4h). Despite this higher density of chloroplasts in the BS of OsCGA1 transactivated lines, we noted that higher variability in the BS chloroplast abundance across different events with many chloroplasts aggregated in the central vacuole and with abundant stromules suggestive of chloroplast degradation intermediates (Figure S10) (Gray *et al*., 2012; Izumi *et al*., 2017). We speculate that since these intermediate structures are rarely observed in wild‐type plants, they were revealed here by artificially enhancing plastid proliferation in the BS. These results suggest that an active chloroplast turnover process is operative in rice BS to maintain low plastid copy numbers (Zhuang and Jiang, 2019).

## Discussion

### The role of CGA1 in photosynthetic gene regulation

The CGA1/GNC gene regulators have long been known to integrate diverse developmental and environmental cues to promote photosynthetic differentiation in *Arabidopsis* (Behringer and Schwechheimer, 2015; Cortleven and Schmülling, 2015). DELLA and light‐mediated degradation of PIF proteins derepress CGA1/GNC1 transcription and promote PEP assembly and plastid gene expression (Yoo *et al*., 2019; Richter *et al*., 2010). In contrast, auxin and GA signalling act to inhibit CGA1/GNC transcription, fine‐tuning cell elongation and greening (Richter *et al*., 2013; Richter *et al*., 2010). The stunted dark green phenotype mediated by *OsCGA1 overexpression* in rice and *Arabidopsis* (Figure S1; Chiang *et al*., 2012; Hudson *et al*., 2013) is reminiscent of the GA signalling mutant, *ga1* and auxin signalling mutant, *arf2* in *arabidopsis (*Richter *et al*., 2010; Richter *et al*., 2013) suggesting that ectopic expression of *CGA1* is sufficient to override endogenous GA and auxin signalling pathways that would otherwise restrict photosynthetic development of the BS. The biogenesis of photosynthetically active chloroplasts is linked to the assembly of plastid‐encoded RNA polymerase and the transcription of photosynthesis‐associated plastid‐encoded genes (PhAPG) in the plastid (Liere and Börner, 2006). Notably, the expression of genes encoding nucleus encoded plastid transcription associated factors and PEP‐associated proteins (PAPs) (Steiner *et al*., 2011) increased in the BS of *pFtGLDp::OsCGA1*. In addition, the increased accumulation of other PhANGs, including genes encoding post‐transcriptional/translational machinery, light harvesting complexes and chlorophyll biosynthetic components, indicated that *OsCGA1* exerts its effect on chloroplast development by regulating nuclear and plastid gene expression. Similar to our observations, Hudson *et al*., 2013 has shown the primary effect of ectopic CGA1 expression is the increased expression of several nuclear‐encoded, chloroplast localized proteins involved in chlorophyll binding and photosynthesis. However, we did not detect the increased expression of genes involved in amino acid biosynthesis, starch biosynthesis and chloroplast division as detected by Hudson *et al*., 2013. This may be due to the differential promoter activities used in the two studies (i.e. a weak vascular‐specific promoter vs. the strong constitutive *ZmUbi1* promoter).

### Engineering orthogonal BS activities

To fine tune the expression of *OsCGA1*, we utilized the well‐characterized vascular‐specific promoter, *FtGLDp*, that was first identified in *Flaveria trinervia* and later characterized in *Arabidopsis thaliana* (Engelmann *et al*., 2008). In rice, we were surprised initially to see strong constitutive GUS staining of the *pFtGLDp::Introns containing GUS* and later realized that removal of introns in the GUS gene cassette that was inserted to enhance the reporter gene expression, conditioned the expected weak vascular‐specific pattern. In similar fashion, the use of an intron‐containing GUS reporter driven by the *Zoyzia Japonica* PEPCK promoter, converts a vascular‐specific to a constitutive leaf expression profile (data not shown; Nomura *et al*., 2005). Introns have often been introduced into reporter constructs to enhance gene expression in plants, especially when used in conjunction with weak promoters in an effort to mitigate transgene silencing and to discriminate reporter gene expression from bacterial expression during *Agrobacterium*‐mediated transformation (Callis *et al*., 1987; Tanaka *et al*., 1990). Though anecdotal, these findings suggest it is necessary to validate tissue‐ and cell‐specific profiles of gene expression across species using GUS reporters. Here, we confirmed the vascular‐specific activity of the *FtGLDp* promoter with LCM transcriptomics and tissue‐specific transactivation of *OsCGA1*.

Our successful dCas9‐mediated transactivation demonstrates the potential of regulating suites of genes in a tissue‐specific manner. In our studies, a single gRNA near the TSS was sufficient to increase expression of *OsCGA1* and the *pOsCGA1*:*:GUS* reporter simultaneously. By multiplexing gRNAs and expressing a dCas9‐activator (with a cell‐specific promoter) it should be feasible to drive multiple Kranz anatomy and/or C_4_ metabolic genes in a cell‐specific manner. Future studies to examine the placement of the gRNA relative to the TSS and multiplexing chimeric dCas9 and transcriptional repressors, such as SRDX or BRD domains could further extend the utility of the dCas9 system (Lowder *et al*., 2015). Similarly, the use of dCas12‐activator/repressor modules would enable the flexibility to simultaneously activate and repress gene expression in the same cell types (Bandyopadhyay *et al*., 2020). Although it was possible to manipulate chloroplast architecture in the BS, we also observed what is likely an increased activity of chloroplast degradation as well. Confocal imaging of BS cells expressing dCas9‐AD fusions revealed a proliferation of stromule projections from chloroplasts and chloroplast accumulation in central vacuoles. These features (increased stromules and vacuolar localization of plastids) can be induced by ABA‐mediated abiotic stress and UV light, respectively (Gray *et al*., 2012; Izumi *et al*., 2017) and are likely intermediate products of chloroplast degradation (Zhuang and Jiang, 2019). Consistent with this hypothesis, rice BS plastid integrity is more sensitive to drought than M plastids of rice (Yamane *et al*., 2003). As CK plays a protective role for the photosynthetic machinery by promoting antioxidant production under high light and dark‐induced senescence (Procházková *et al*., 2008; Zavaleta‐Mancera *et al*., 2007), it is likely that ABA‐ and CK‐mediated responses act antagonistically in the BS. In addition to ABA, protoxylem and procambium are rich in the phytohormones GA and auxin, respectively, which as discussed above may also inhibit the transcription of CGA1/GNC around vasculature (Scarpella *et al*., 2006; Yamazaki *et al*., 2018).

### Engineering C_4_ activities in rice

In the context of engineering C_4_ activities into rice this study provides both promise and new challenges. A clear challenge in engineering C_4_ activities is the lack of BS‐specific promoters (Engelmann *et al*., 2008). To date only a single vascular‐specific promoter has been conclusively identified that can drive vascular‐specific expression, despite intensive investigations (Schuler *et al*., 2016). Here, we demonstrate that a single promoter can be used to drive expression from at least two genes. Furthermore, we demonstrate that a single gRNA is sufficient to drive ectopic gene expression, thus, in theory, dozens of genes could be co‐regulated using a single promoter driving expression of a dCas9‐AD by simply designing a tandem array of gRNA sequences targeted to the promoter regions of endogenous or transgenically delivered genes. A previous study suggested a minimum of 20 genes will need to be engineered in both BS and M cells to install a minimal C_4_ circuit in rice (Peterhansel, 2011). The dCas9 technology detailed here is one such enabling technology to realize this potential. Although the focus of this work was on the manipulation of the CGA1 network, work by Langdale and colleagues has suggested that BS plastid area could also be increased through the ectopic expression of *GOLDEN2* and *GOLDEN2‐like* (Wang *et al*., 2017). Future studies to examine the potential for selectively increasing plastid numbers in the BS together with the BS‐specific expression of either CGA1 or GLK could result in additive effects that would increase both plastid size and number. However, this study also suggested the presence of a BS plastid degradation pathway that serves to limit the proliferation of BS chloroplasts in rice. Thus, to engineer a photosynthetic BS in rice, it may also be necessary to manipulate this autophagic response to maintain plastid integrity.

## Material and Methods

### Generation of constructs

All constructs were generated using the Golden Gate cloning system (Engler *et al*., 2014; Weber *et al*., 2011). Full‐length cDNA of OsCGA1 (Gene bank Accession No. AK099607) was obtained from NIAS DNA Bank (http://www.dna.affrc.go.jp/). Fragments were amplified by PCR and subcloned into pICH41308 using BbsI digestion and ligation, to domesticate (removal of internal BbsI and BsaI restriction sites). The *Flaveria trinervia* Glycine Decarboxylase p‐subunit promoter was received from Dr. Udo Gowik and was domesticated using PCR (Engelmann *et al*., 2008). For the dCas9‐AD module, dCas9 and two tandemly linked activation domains, VP64‐EDLL, were domesticated from pYPQ176 (Lowder *et al*., 2018). The gRNA expression modules from pOsU6snRNApol and pAtU6snRNApol were also domesticated from pYPQ141A and pYPQ141C. A 2126 bp upstream sequence of OsCGA1 was isolated from Kitaake gDNA and domesticated into the pL0M‐PU module. The gRNA sequences were designed using two web‐based applications (https://crispr.dbcls.jp/ and http://crispor.tefor.net/) and selected based on the proximity to the transcriptional start site of the OsCGA1 promoter. For the gRNA cloning, forward primer and complementary primer are annealed and cloned into the corresponding gRNA modules with appropriate positions of L1 modules using Esp3I and Eco31I. All constructs were generated via Golden Gate assembly using Thermo FastDigest restriction enzyme and T4 DNA Ligase (Thermo Fisher Scientific, Waltham,USA). All L0 module insert sequences, primer sequences for gRNA cloning and layout of assemblies used in this study are summarized in Table S4. All basal L0 modules and gRNA expression modules are cloned in the appropriate position of backbone and their insert sequence is provided between 4 bp overhangs in Table S4. Constructs received form ENSA projects were marked as EC*****. For L1 constructs and L2 constructs are assembled with the presented modules of lower order in Table S4.

### Rice transgenic production

*Agrobacterium*‐mediated transformation of rice var. Kitaake was developed based on a previous protocol for Nipponbare (Ozawa, 2009) with major modifications in media composition and light conditions. Kitaake rice seeds were dehusked and sterilized with a 50% bleach solution for 30 min. Seeds were rinsed three times with sterile water and incubated in a 1% hydrogen peroxide solution for 1 h. Surface‐sterilized seeds were then placed on MSD (1x Murashige Skoog Basal salts (PhytoTechnology), 1x B5 Gamborg’s vitamins, 0.03% casamino acids, 0.11% L‐proline, 4% Maltose, 1 mg/mL 2,4‐D) with 0.3% gelrite to induce callus under continuous light at 28 °C for 2 weeks. Agro strain LBA4404 carrying the constructs was inoculated on AB liquid media (Wise *et al*., 2006) and resuspended in AAM media (Hiei *et al*., 1994) with 15 ųg/ml of acetosyringone. Calli were submerged in an agrobacterium suspension and blotted dry on sterile paper to remove excess Agrobacteria. Co‐cultivation was performed on three sheets of No. 2 filter papers (Advantec, JAPAN) wetted with MSD liquid supplemented with 0.01% L‐Cysteine, 0.5% glucose and 40 μg/mL of acetosyringone at 22 °C under dark for 3 days. Co‐cultivated tissues were washed with sterile water three times and 200 μg/mL timentin‐containing water. The washed calli were selected on the 0.3% gelrite MSD with hygromycin B (50 μg/mL) and timentin (100 μg/mL) for 2‐3 weeks. Newly arising nodal calli were transferred to MSR media (1× MS salts, 1× B5 Gamborg vitamin, 4% maltose, 0.2% casamino acid, 0.1 μg/mL NAA and 2 μg/mL Kinetin) with hygromycin B (50 μg/mL) and timentin (100 μg/mL) and 0.4% gelrite under continuous light at 28 °C. Regenerated explants with intact roots and shoots were transferred to soil under greater than 50% relative humidity to recover.

### Plant growth

Plants were grown for seed propagation and phenotypic characterizations at the Donald Danforth Plant Science Center, St. Louis, MO under greenhouse growth conditions. Rice *var*. Kitaake and derived transgenic were grown in turface (Field & Fairway, PROFILE products LLC) in 175 mL square pots in flooded trays. Plants were given intermittent fertilizer with 200 ppm N. of 15–16–17 peat‐lite (ICL Special Fertilizers, OH) as needed. The greenhouse was maintained under daily photoperiod of 14 h, with radiation incident on the plant canopy composed by external sun radiation plus radiation emitted by supplemental HID lights mixed with Metal Halide and High Pressure Sodium lamps (the latter with relative intensity of 7: 3). In the time window from 10 a.m. to 2 p.m. standard time, the Photosynthetic Photon Flux Density (PPFD) incident on the plant canopy was in the range of 800–1000 µmol photons m^−2^ s^−1^. The air temperature was set at 28/25 °C during the day/night, respectively. Plants were given intermittently with fertilizer of 200 ppm N. of 15‐16‐17 peat‐lite (ICL Special Fertilizers, OH) as needed.

Plants used for leaf photosynthetic analysis were grown in a controlled environment growth chamber (Bigfoot series, BioChambers Inc., Winnipeg, MB, Canada) at the School of Biological Sciences at Washington State University, Pullman, WA (USA). The growth chamber was set with a daily photoperiod of 14 h with a maximum PPFD of 600 µmol photons m^−2^ s^−1^ incident on the plant canopy, air temperature of 26 °C and air relative humidity of ~70% (corresponding to air vapour pressure deficit of ~1.6 kPa); during the dark period air temperature was set at 22 °C. Plants were grown individually in 7.5*‐*L free drainage pots in a Sunshine Mix LC‐1 soil (Sun Gro Horticulture, Agawam, MA) mixed with turface (ratio of 3:1 in volume) under daily irrigation and fertilized twice a week to pot saturation.

For LCM analysis, plants were grown in 1:1 mixture of topsoil and sand for about 2 weeks in a growth room with a daily 12 h photoperiod, and PPFD of 300 μmol photons m^−2^ s^−1^ incident on the plant canopy. Air temperatures were set at 28 and 25 °C during the photoperiod and in the dark, respectively, and air relative humidity was 65% (corresponding to maximum air vapour pressure deficit of ~1.9 kPa).

### RNA extraction and RT‐qPCR analysis

For the *pFtGLDP::OsCGA1* transgene expression (Figure 1b), 2 cm of mid and tip regions of emerging fourth leaves were harvested into round‐bottom microfuge tubes and frozen in liquid N_2_. For dCas9‐mediated OsCGA1 activation, 2 cm sections of the mid region of the leaf were used (Figure 4g). Frozen tissues were ground by bead beating in a paint shaker with liquid N_2_. RNA was extracted using TriPure isolation reagent (Roche Diagnostics, Basel, Switzerland) and 1 μg RNA of individual samples were used for residual gDNA removal using RQ1 RNase‐Free DNase (Promega, Madison, USA). After ethanol precipitation, DNase‐treated RNA was used for cDNA synthesis using Improm‐II™ reverse transcriptase (Promega) and Oligo(dT)15 primer (promega) according to the manufacturer’s instructions. cDNA was used for the RT‐qPCR using a LightCycler®️ 480 Green I Master mix (Roche Diagnostics) and primer set (Table S3) for target and reference genes on LightCycler 480 II qPCR machine (Roche Diagnostics). Os14‐4‐3E (LOC_Os11g34450) were used for normalization in Figure 1b and Os14‐3‐3E was used solely for the normalization for Figure 4g. Normalized and relative expression were calculated based on the methods in (Taylor *et al*., 2019).

### LCM sample preparation

The middle 1‐cm section of the third expanded leaf was sampled at 4 h after dawn and fixed in ice‐cold 100% acetone for 4 h on ice. The leaf tissue for LCM was then processed as described (Hua and Hibberd, 2019). In brief, leaf tissue was dehydrated through a series of ethanol gradients (v/v) of 70%, 85%, 95% and 2*100%, then kept in 100% ethanol overnight; the next day, leaf tissue was infiltrated with 25%, 50%, 75% and 2*100% Steedman’s wax in ethanol at 40 °C for 2 h each, then the leaf was infiltrated with 100% Steedman’s wax overnight, the next day, tissue was embedded in petri‐dish.

### Laser capture microdissection and RNA extraction

Paradermal sections of 7 µm thickness were prepared with a Reichert‐Jung microtome and mounted on PEN membrane glass slides (Applied Biosystems) with DEPC‐treated water. Steedman’s wax was removed by incubating slides in 100% acetone for 1 min, then Laser capture microdissection was performed on ArcturusXT LCM System (Applied Biosystems) following the user manual. Bundle sheath strands and mesophyll cells were harvested on CapSure Macro LCM Caps (Applied Biosystems), RNA was extracted using PicoPure RNA Isolation Kit with on‐column DNase treatment according to manufacturer's instructions. RNA quality was examined using Bioanalyzer 2100 (Agilent, Santa Clara) in combination with RNA pico chip.

### LCM Library preparation and sequencing

From 37 to100 ng bundle sheath strand (BSS) and M LCM isolated RNA from lines #12‐2‐4 and #12‐4‐4 were used for 3’ mRNA‐seq library preparation using a QuantSeq 3’ mRNA‐Seq Library Prep Kit (Lexogen, Vienna, Austria) according to the manufacturer's instructions. The RNA integrity number (RIN) values ranged from 5.6–6.7 as determined by analysis of electropherogram output from Bioanalyzer (Agilent). Sixteen PCR cycles were used to enrich libraries and libraries were sequenced using 75‐bp single end reads on an Illumina NextSeq 550 platform.

### Confocal imaging and quantification of chloroplast volume

Corresponding fourth or fifth leaf segments were immersed and fixed for 30min in the freshly made 4% paraformaldehyde (80 mM sodium phosphate buffer, 0.1% Triton X‐100) with vacuum infiltration. After vacuum infiltrations, leaf tissue was placed in fixative for 1 ~ 2 h, rinsed and then stored in 0.1 M EDTA solution. Fixed tissues were stored in darkness at 4 °C and imaged within 6 months. Stored tissues were embedded in 7% low melting agarose and 50 μm sections generated with a vibratome. Both transverse and longitudinal sections were placed in a 5 μM calcofluor white solution for the cell wall imaging. Sections were observed with a Leica TCS SP8 confocal laser scanning microscope using a HC PL APO CS2 40×/1.10 WATER objective lens (Leica Microsystems, Manheim, Germany). Sections were imaged using a 405 nm UV laser for excitation. Calcofluor white and chlorophyll autofluorescence were obtained with the 460–480 nm and 680–700 nm band emission spectrum, respectively, with a 16 line average resolution. For chloroplast volume quantification, 90 nm thick optical section images were taken for each sample with fixed voxel size with pixel size 90 nm × 90 nm for the consistency. Optical stacks were trimmed and deconvoluted using Huygens Essential software (Scientific Volume imaging, Hilversum, Netherland). Chloroplast volumes were obtained by surface rendering on the IMARIS (Oxford instruments, UK) and chloroplasts within individual BS cells were manually picked based on the calcofluor staining. Longitudinally sliced BS cells were considered as partial cylinder and volume of each BS cells were roughly estimated by

$\text{Volume}=L(R^{2}\cos^{‐1\left(\frac{R‐D}{R}\right)}‐\left(R‐D\right)\sqrt{2RD‐D^{2}}$ measuring length (*L*), radius (*R*) and depth (*D*) of the cylinder.

### Transmission electron microscopy

Leaf 4 was prepared for transmission electron microscopy (TEM), quantification of cellular features, and immunodetection of photosynthetic enzymes and glycine decarboxylase was previously described (Khoshravesh *et al*., 2017). Leaf sections from the middle of recent fully expanded leaves were fixed in 1% glutaraldehyde and 1% paraformaldehyde in 0.1 M sodium cacodylate buffer (pH 6.8) overnight at room temperature. Samples were subsequently dehydrated in ethanol: H2O with 10% increment increases of 10% to 100% ethanol for 1 h at each incremental increase. This was followed by two 1 h incubations in 100% ethanol before the tissue was infiltrated in London Resin White (LRW) using 1:3, 1:1, and 3:1 ratio of LRW to 100% ethanol (8 h each increment), followed by 2 × 100% LRW (8 h each). LRW infiltrated leaf samples were polymerized at 60 °C in an oxygen‐free environment for 12 h. Sections of 50–70 nm were collected for immunolocalization and TEM imaging. Images for TEM were captured on a Phillips 201 transmission electron microscope equipped with an Advantage HR camera system (Advanced Microscopy Techniques). For quantification of organelle numbers and area from EM images, Kruskal–Wallis one‐way analysis of variance was performed, followed by a Dunn's *post hoc* test in R.

### Immunohistochemistry

Immunohistochemistry was performed as detailed in (Wang *et al*., 2017). In brief, 50–70 nm sections of LRW embedded tissue were rehydrated in 0.01 M phosphate saline buffer (PBS) pH 7.4, blocked for 20 min in 0.5% bovine serum albumin (BSA) in PBS, and then rinsed in PBS 3× for 15 min before incubating for 3 h in primary antibody at the following concentrations: 1:50 (anti‐glycine decarboxylase), 1:100 (anti‐RuBisCo and anti‐RuBisCo activase) and 1:400 (anti‐FBPase) in 0.1% BSA/PBS. Sections were then rinsed in PBS 3 × 15 min before incubation with secondary antibody (18 nm Colloidal Gold‐AffiniPure Goat Anti‐Rabbit IgG) for 1 h at a concentration of 1:20 (glycine decarboxylase) or 1:40 (photosynthetic enzymes) in 0.1% BSA/PBS. Samples were then rinsed in PBS three times for 15 min each, followed by ultrapure water (3 × 15 min) before being stained with 4% uranyl acetate for 10 min, and then lead citrate for 5 min (Reynolds, 1963).

### RNA seq data analysis

Raw RNAseq reads were quality checked using FastQC (https://github.com/s‐andrews/FastQC). Adapter, quality trimming, and polyA tail removal was performed using TrimGalore (https://github.com/FelixKrueger/TrimGalore) with the following parameters (stringency 3, clip_R1 13, length 20, q 20). Gene annotation and transcript sequences (Osativa_323_v7.0) were obtained from Phytozome11. Transcript quantification and read counts were performed using Salmon (quasi‐mapping based, parameters: incompatPrio 0.0, noLengthCorrection, libType SF) (Patro *et al*., 2017). Read counts were imported with tximport R‐package (Soneson *et al*., 2015). Differential expression analysis was carried out with the edgeR package (FDR < 0.10) (Robinson *et al*., 2010).

## Competing interests

The authors have no competing interests to declare.

## Author contributions

D‐Y.L. conducted molecular genetics experiments and confocal imaging, L.H. performed LCM analysis, R.K. performed EM analysis and immunohistology, R.G. performed photosynthetic assays, I.K. conducted RNA seq analysis, A.C. analysed photosynthetic measurements, T.L.S. analysed EM and immunohistochemistry data, J.H. analysed LCM data, D‐Y.L and T.P.B conceptualized the project and wrote the manuscript. All authors contributed to editing the manuscript.

## Materials & Correspondence

Please contact Dr. Thomas Brutnell (tom@viridisgenomics.com) for requests of materials and correspondence. Data is available through a CC BY license agreement. RNAseq data is accessible through NCBI accessions PRJNA736666 and PRJNA739378.

## Supporting information


**Figure S1**. Overexpression analysis of *OsCGA1* in *Kitaake var*. rice.

**Figure S2**. *Flaveria trinervia Glycine Decarboxylase p‐subunit* (FtGLDp) promoter activity and chloroplast proliferation in the BS of the *pFtGLDp::OsCGA1* transgenic lines.

**Figure S3**. Accumulation of photosynthetic enzymes in WT and Transgenic lines.

**Figure S4**. Leaf photosynthetic and biochemical responses

**Figure S5**. Summary of leaf gradient RNA seq in two independent events of *pFtGLDP::OsCGA1*.

**Figure S6**. Microdissection images of BS strands and M strands and their RNA profiles from *pFtGLDP::OsCGA1* transgenic and nulls.

**Figure S7**. Pageman analysis using DE genes in BSS LCM seq.

**Figure S8**. Transcriptional activation test on *OsCGA1 promoter GUS* reporter using dCas9‐mediated transactivation in heterologous system *Nicotiana tabaccum*.

**Figure S9**. Tissue specific expression of *pOsCGA1::GUS* reporter by dCas9 mediated activation in *Kitaake* transgenics.

**Figure S10**. Chloroplast morphologies in the bundle sheath cells of *dCas9* activation lines.

**Table S1**. TEM and quantitative measurements of organelle phenotypes in *pFtGLDp::OsCGA1* transgenic.

**Table S2**. Summary of transgenic lines used in this study.

**Table S3**. Primer and taqman probe sequences used in this study.

**Table S4**. Golden gate construct assembly and sequence information.

**Table S5**. DEGs in the BS LCM seq between pFtGLDp::OsCGA1 transgenic and their null (FDR<0.05).

**Supplementary Material**.
