# Supplementary figures and images for: Engineering chloroplast development in rice through cell‐specific control of endogenous genetic circuits
Source: Plant Biotechnol J. 2021 Aug 18;19(11):2291–303. doi: 10.1111/pbi.13660 (PMC8541780; doi:10.1111/pbi.13660)

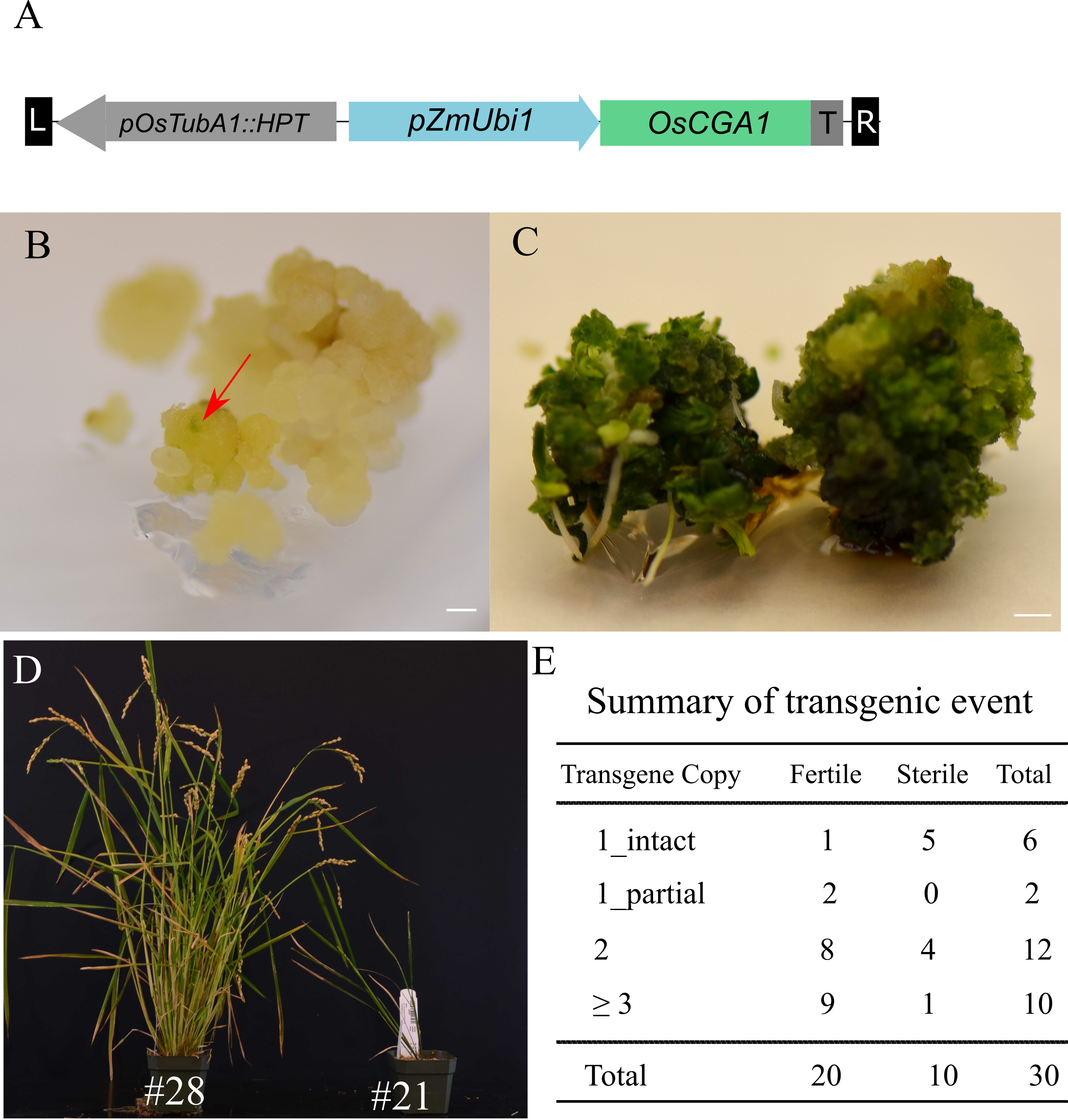

Supplement: Supplementary file 1 — Figure S1. Overexpression analysis of OsCGA1 in Kitaake var. rice. [file PBI-19-2291-s014.PNG]

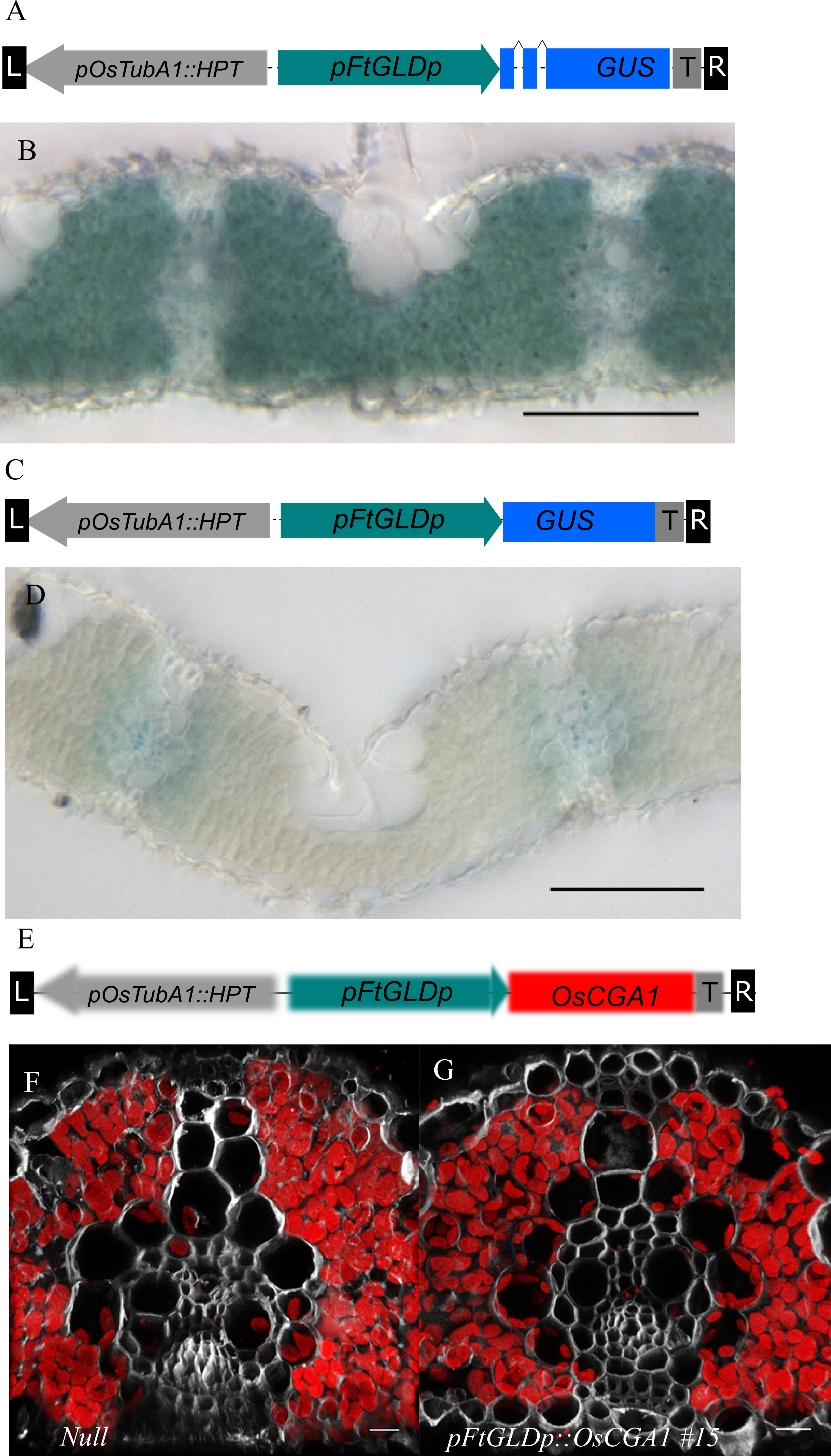

Supplement: Supplementary file 2 — Figure S2. Flaveria trinervia Glycine Decarboxylase p‐subunit (FtGLDp) promoter activity and chloroplast proliferation in the BS of the pFtGLDp::OsCGA1 transgenic lines. [file PBI-19-2291-s009.png]

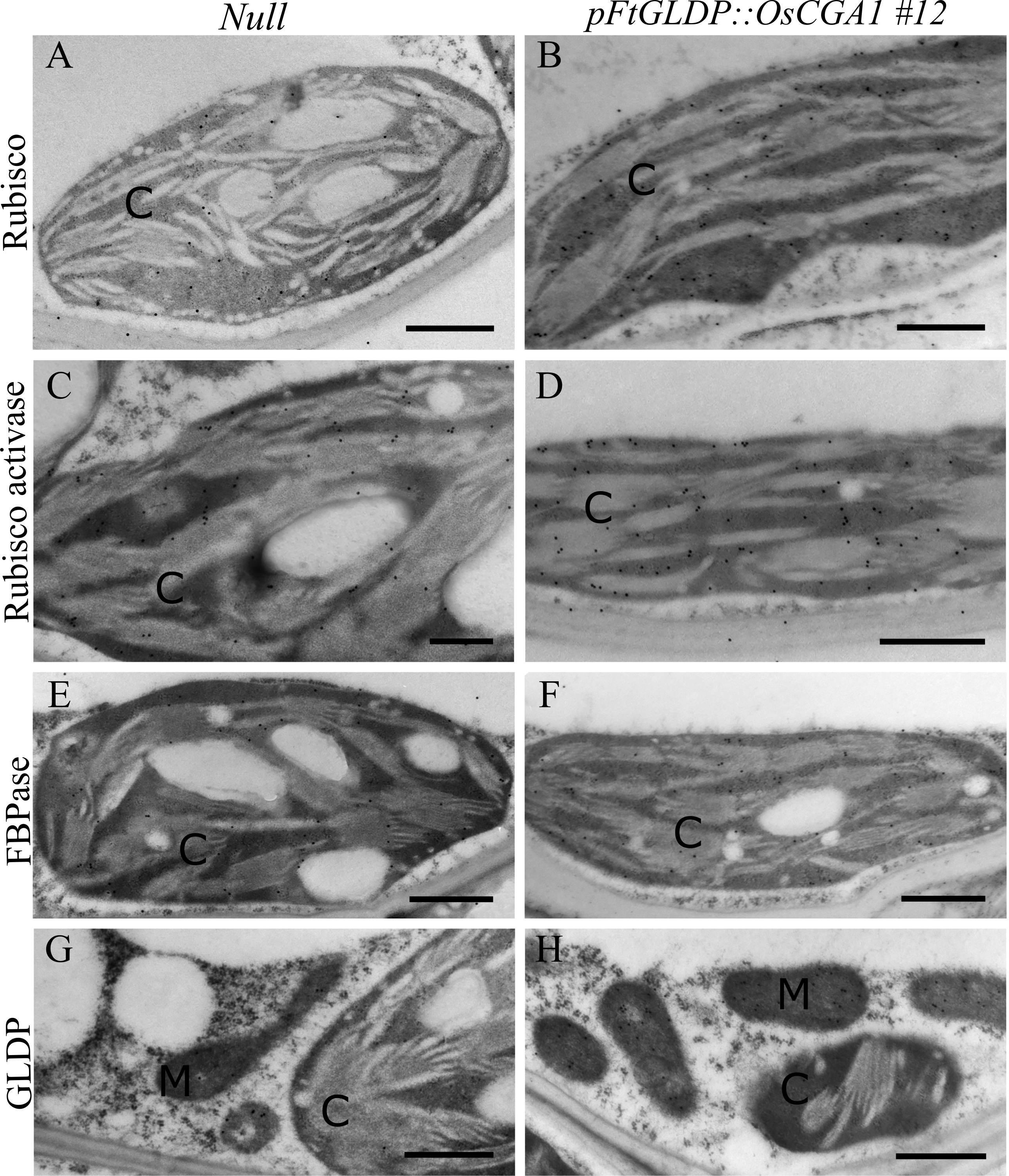

Supplement: Supplementary file 3 — Figure S3. Accumulation of photosynthetic enzymes in WT and Transgenic lines. [file PBI-19-2291-s005.png]

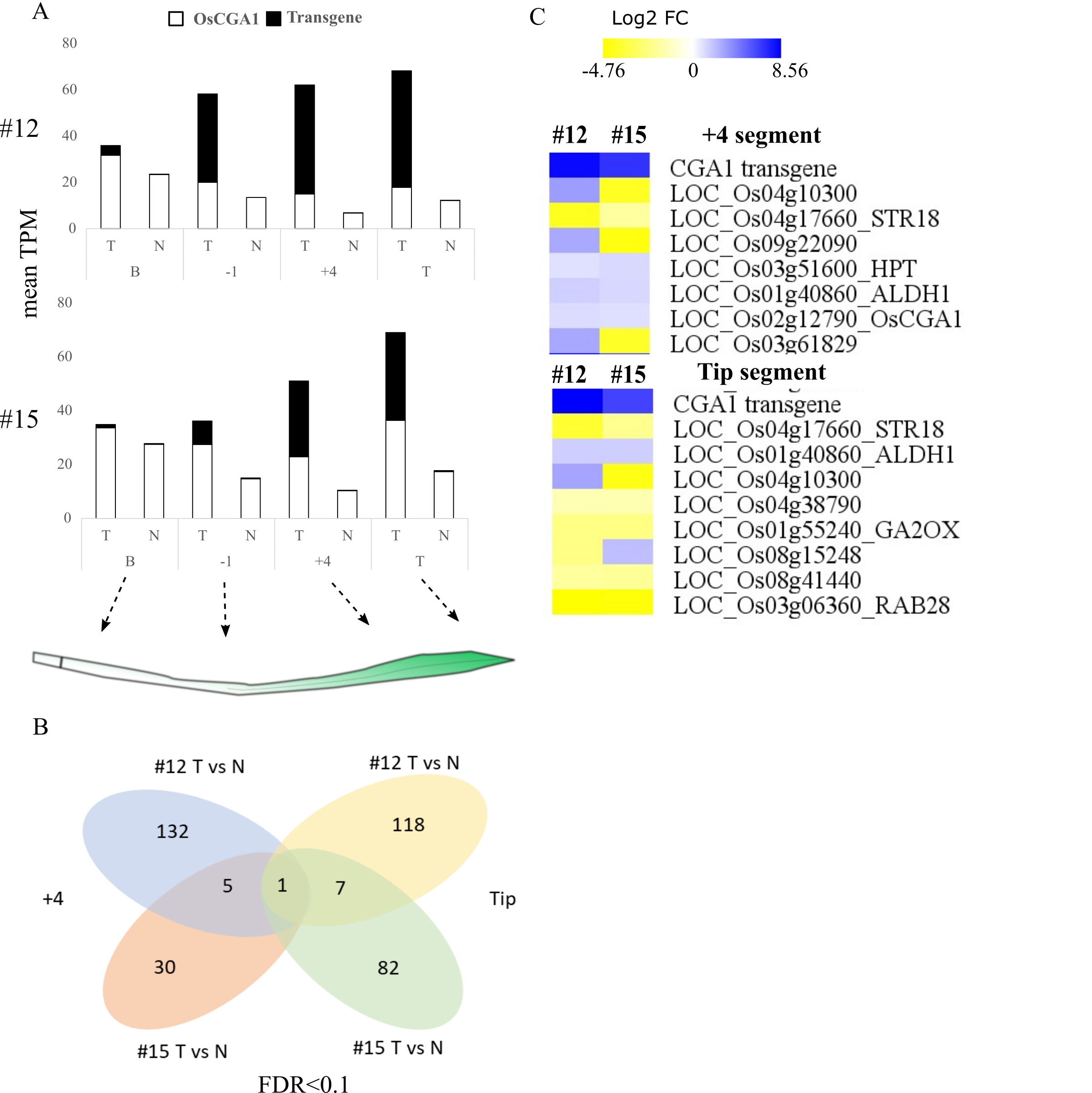

Supplement: Supplementary file 5 — Figure S5. Summary of leaf gradient RNA seq in two independent events of pFtGLDP::OsCGA1. [file PBI-19-2291-s004.png]

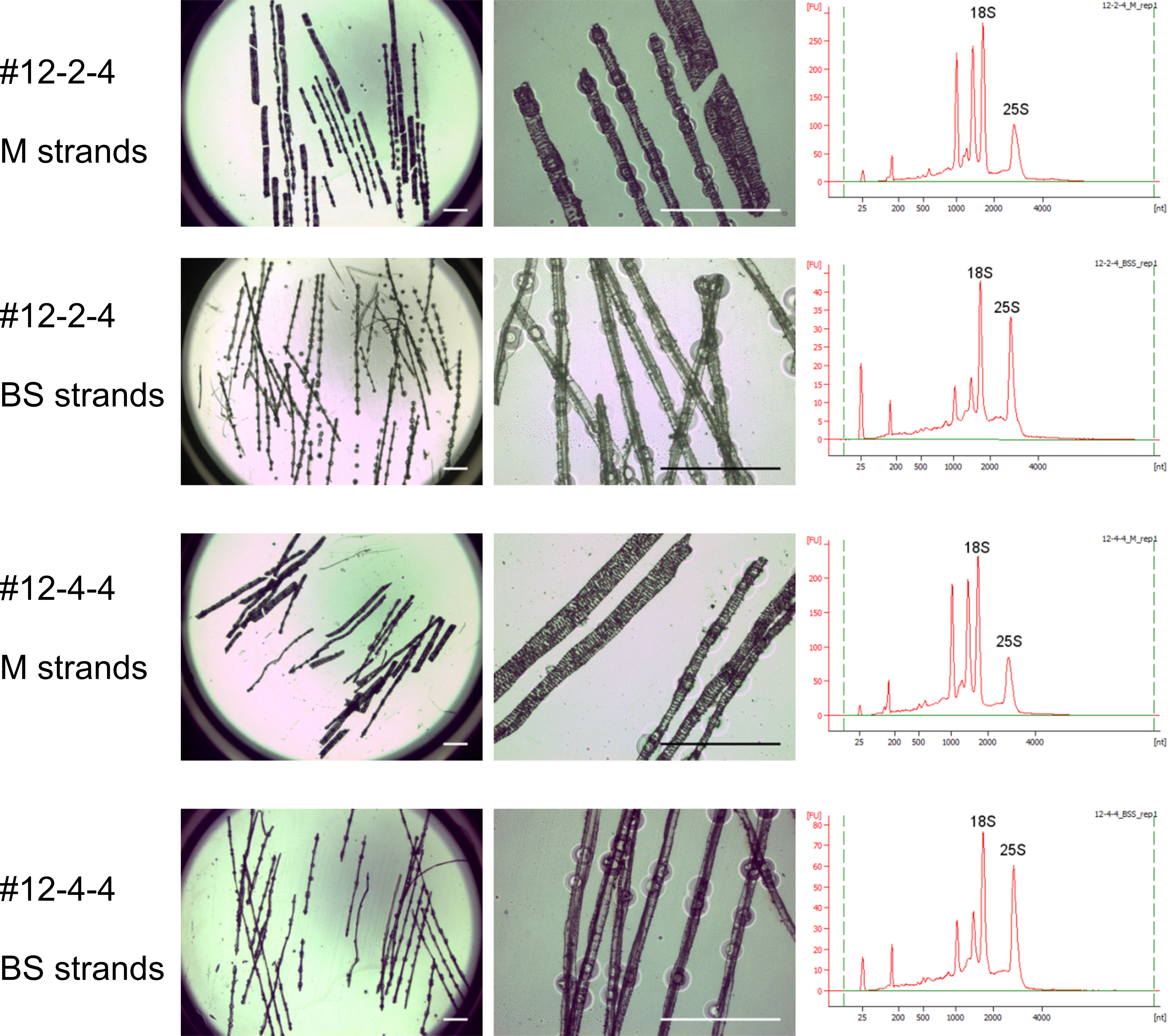

Supplement: Supplementary file 6 — Figure S6. Microdissection images of BS strands and M strands and their RNA profiles from pFtGLDP::OsCGA1 transgenic and nulls. [file PBI-19-2291-s013.png]

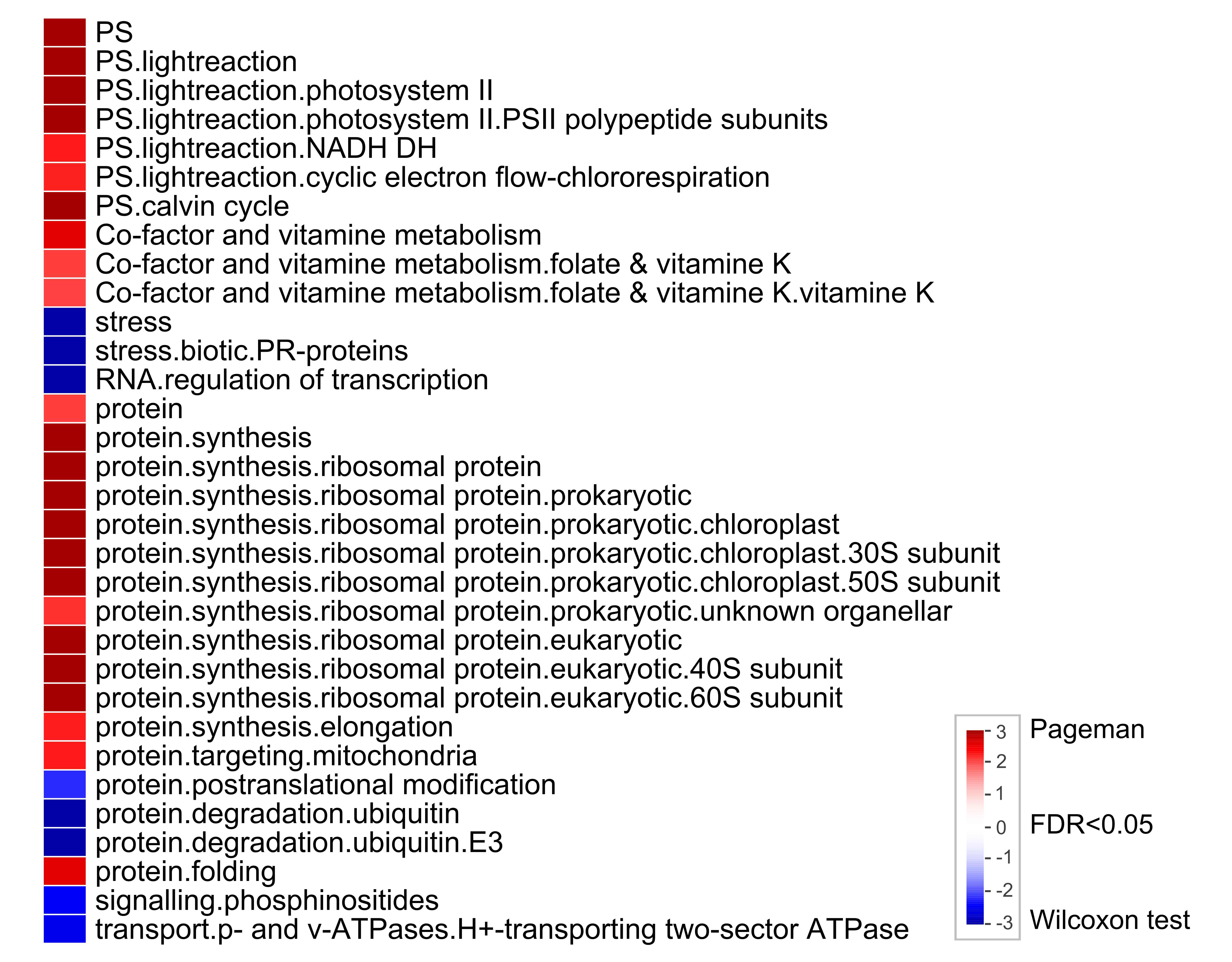

Supplement: Supplementary file 7 — Figure S7. Pageman analysis using DE genes in BSS LCM seq. [file PBI-19-2291-s016.png]

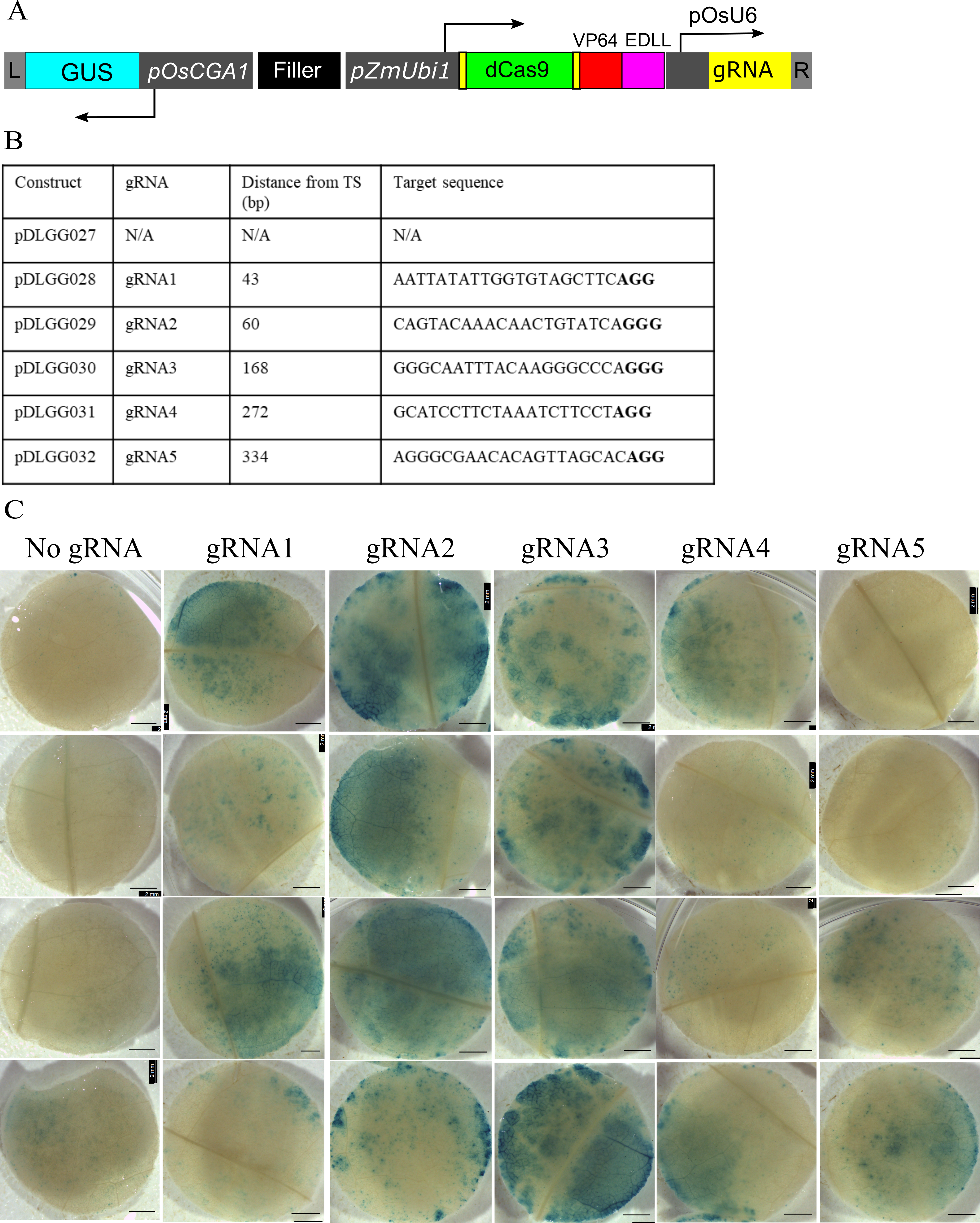

Supplement: Supplementary file 8 — Figure S8. Transcriptional activation test on OsCGA1 promoter GUS reporter using dCas9‐mediated transactivation in heterologous system Nicotiana tabaccum. [file PBI-19-2291-s001.png]

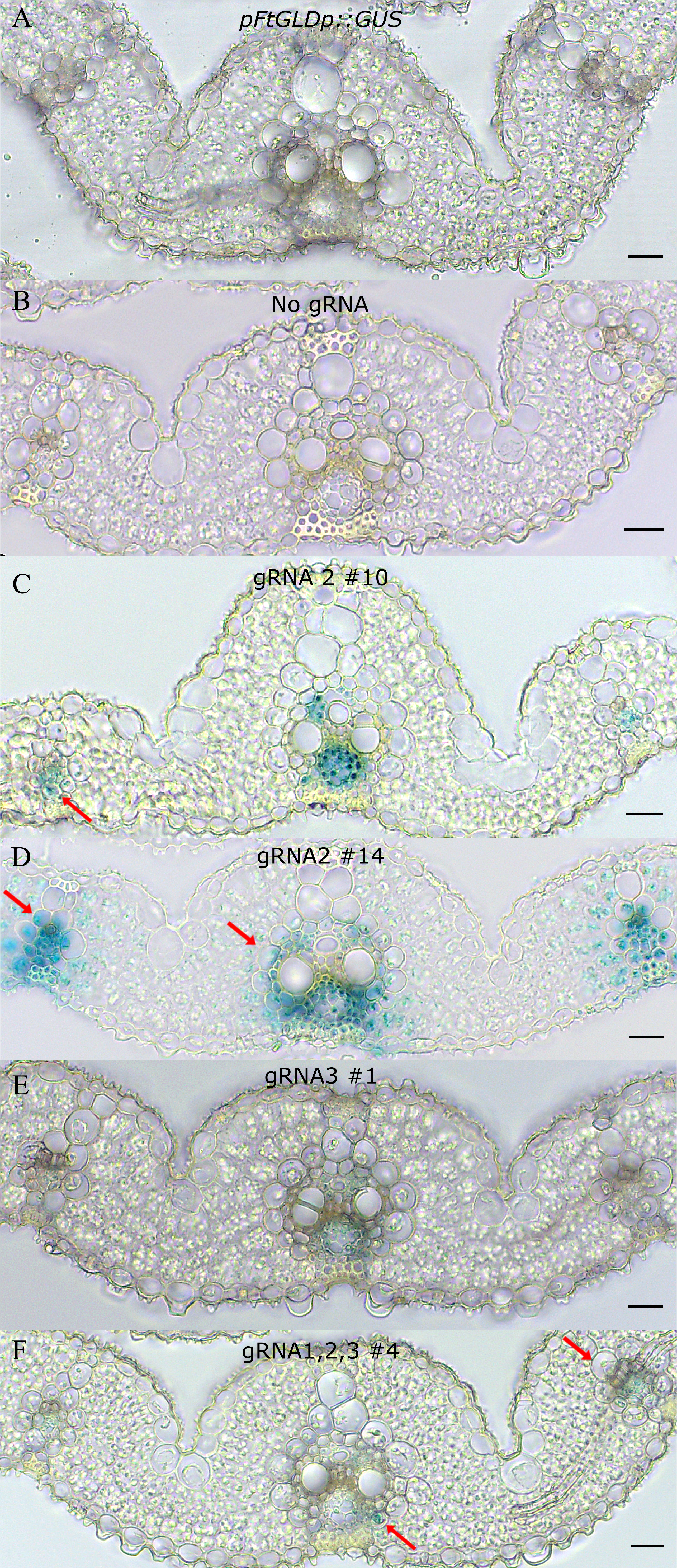

Supplement: Supplementary file 9 — Figure S9. Tissue specific expression of pOsCGA1::GUS reporter by dCas9 mediated activation in Kitaake transgenics. [file PBI-19-2291-s012.png]

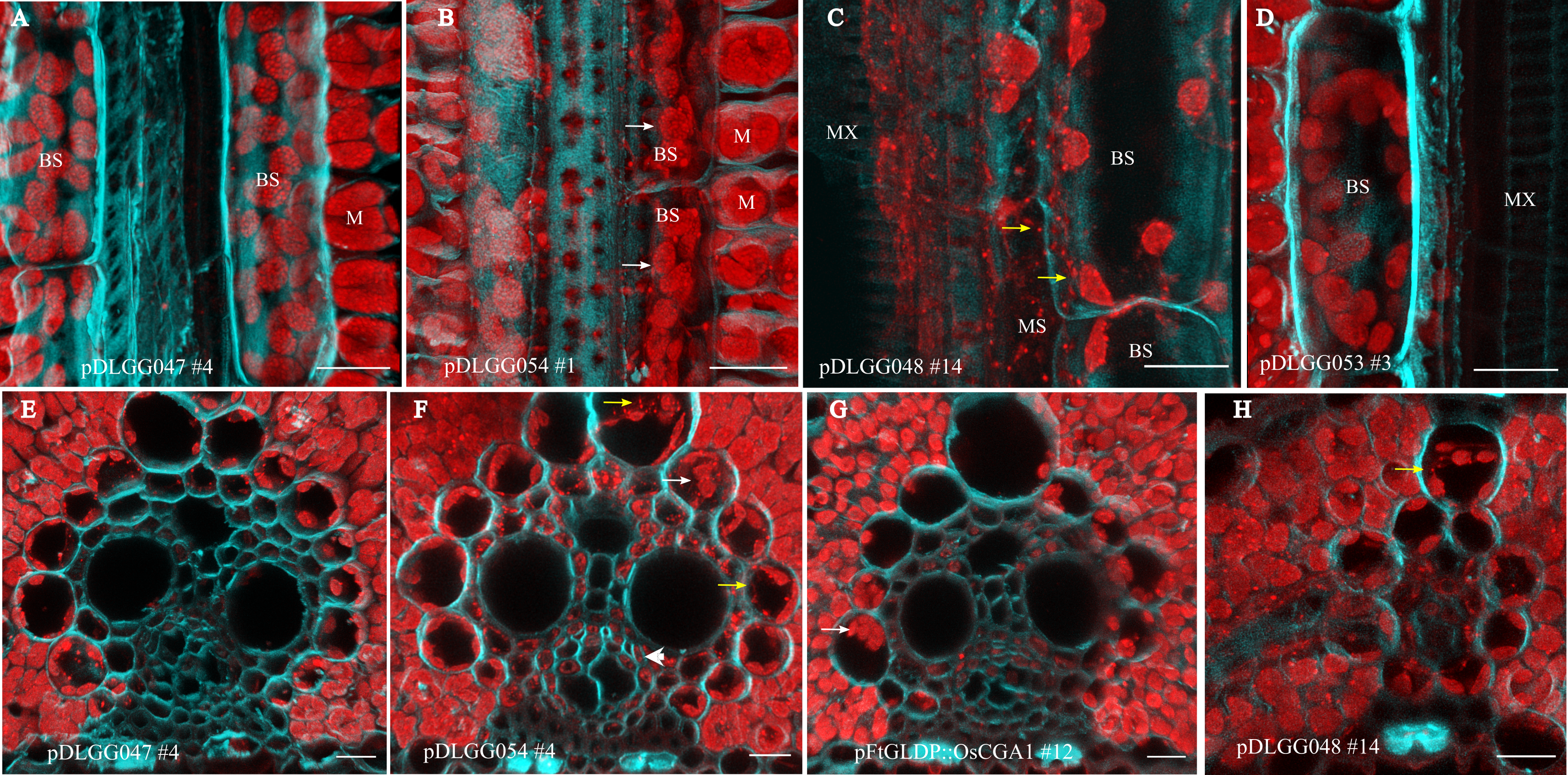

Supplement: Supplementary file 10 — Figure S10. Chloroplast morphologies in the bundle sheath cells of dCas9 activation lines. [file PBI-19-2291-s010.png]
