# Supplementary material for: Engineering chloroplast development in rice through cell‐specific control of endogenous genetic circuits
Source: Plant Biotechnol J. 2021 Aug 18;19(11):2291–303. doi: 10.1111/pbi.13660 (PMC8541780; doi:10.1111/pbi.13660)
Supplement: Supplementary file 11 — Table S1. TEM and quantitative measurements of organelle phenotypes in pFtGLDp::OsCGA1 transgenic. [file PBI-19-2291-s011.docx]

|  |  | *Null* | *pFtGLDp::OsCGA1* | | |
| --- | --- | --- | --- | --- | --- |
|  |  | #12-17 | #2-13 | #12-15 | #15-19 |
| Mitochondria planar area/Planar cell area, % | BS | 0.49 ± 0.6^a^ | 0.37 ± 0.2^a^ | 0.36 ± 0.2^a^ | 0.82 ± 0.7^b^ |
| Mitochondria number /Planar cell area, µm^2^×10^-3^ | BS | 28.7 ± 26.9^a^ | 25.4 ± 15.6^a^ | 24.2 ± 15.2^a^ | 44.0 ± 32.6^b^ |
| Peroxisome planar area/Planar cell area, % | BS | 0.13±0.3^a^ | 0.10±0.3^a^ | 0.18±0.4^a^ | 0.27±0.5^a^ |
| Peroxisome number /Planar cell area, µm^-2^ ×10^-3^ | BS | 2.8±5.3^a^ | 3.4±6.4^a^ | 4.1±6.9^a^ | 4.2±1.0^a^ |

**Supporting Table 1. TEM and quantitative measurements of organelle phenotype in *pFtGLDp::OsCGA1* transgenic**

Quantification of planar area of organelles in bundle sheath cells of WT and transgenic lines. Values are mean ± SD (n = 3 [10 -15 cells/3 individuals]). ^a-d^ values with the same letters represent no significant difference (p > 0.05) by Kruskal Wallis one way analysis of variance followed by a Dunn's test.

*These values are quantified from two biological replicates.
