# Supplementary material for: Engineering chloroplast development in rice through cell‐specific control of endogenous genetic circuits
Source: Plant Biotechnol J. 2021 Aug 18;19(11):2291–303. doi: 10.1111/pbi.13660 (PMC8541780; doi:10.1111/pbi.13660)
Supplement: Supplementary file 16 — Supplementary Material. [file PBI-19-2291-s002.docx]

**Supplementary Materials for**

**Engineering chloroplast development in rice through cell-specific control of endogenous genetic circuits**

Dong-Yeon Lee^1^, Lei Hua^2^, Roxana Khoshravesh^3,4^, Rita Giuliani^5^, Indrajit Kumar^1^, Asaph Cousins^5^, Tammy Sage^3^, Julian M. Hibberd^2^ and Thomas P. Brutnell^1,6,7*^

*Correspondence to Dr. Thomas Brutnell ([tom@viridisgenomics.com](mailto:tom@viridisgenomics.com))

**This file includes:**

**Supplementary Figure legends**

Supplementary Figure 1. Overexpression analysis of *OsCGA1* in *Kitaake var.* rice

Supplementary Figure 2. *Flaveria trinervia Glycine Decarboxylase p-subunit* (FtGLDp) promoter activity and chloroplast proliferation in the BS of the *pFtGLDp::OsCGA1* transgenic lines

Supplementary Figure 3. Accumulation of photosynthetic enzymes in WT and Transgenic lines

Supplementary Figure 4. Leaf photosynthetic and biochemical responses

Supplementary Figure 5. Summary of leaf gradient RNA seq in two independent events of *pFtGLDP::OsCGA1*

Supplementary Figure 6. Microdissection images of BS strands and M strands and their RNA profiles from *pFtGLDP::OsCGA1* transgenic and nulls

Supplementary Figure 7. Pagemen analysis using DE genes in BSS LCM seq.

Supplementary Figure 8. Transcriptional activation test on *OsCGA1* *promoter GUS* reporter using dCas9-mediated transactivation in heterologous system *Nicotiana tabaccum*

Supplementary Figure 9. Tissue specific expression of *pOsCGA1::GUS* reporter by dCas9 mediated activation in *Kitaake* transgenics

Supplementary Figure 10. Chloroplast morphologies in the bundle sheath cells of *dCas9* activation lines

Supplementary Table 1. TEM and quantitative measurements of organelle phenotype in *pFtGLDp::OsCGA1* transgenic

**Supplementary Methods**

**References**

**Other Supporting Materials for this manuscript include the following:**

Supplementary Table 2. Summary of transgenic lines used in this study.

Supplementary Table 3. Primer and taqman probe sequences used in this study.

Supplementary Table 4. Golden gate construct assembly and sequence information.

Supplementary Table 5. DEGs in the BS LCM seq between pFtGLDp::OsCGA1 transgenic and their null (FDR<0.05)

**Supplementary Figure Legends**

**Supplementary Figure 1. Overexpression analysis of *OsCGA1*** **in *Kitaake var.* rice**

(**A**) Construct scheme for the constitutive expression of *OsCGA1*. The maize *Ubiquitin1* promoter was used to drive the *OsCGA1* transgene and *OsTubA1* promoter-Hygromycin Phosphotransferase (HPT) cassette used for plant selection [(Jeon *et al.*, 2000)](https://paperpile.com/c/PATKsO/xU9j). T, nos terminator; L, Left border of transfer DNA; R, right border of T-DNA. (**B**) Occasional greening (red arrowhead) of resistant calli during the hygromycin selection stage. (**C**) dark green arrested calli of *OsCGA1* OX explants on standard regeneration media with kinetin. These arrested calli were rescued on kinetin-free media but plants were severely stunted and sterile. (**D**) pleiotropic effects induced by *OsCGA1* overexpression. Most single copy insertional events (right) displayed a dark green leaf, stunted growth, and complete sterility compared to the multi copy transgenic events (left). #28, *OsCGA1* OX event #.28; #21, *OsCGA1* OX event # 21. (**E**) summary of transgenic events. Most events showing a dark-green phenotype contained 1~2 T-DNA copies and were sterile. Scale bar, 1mm.

**Supplementary Figure 2. *Flaveria trinervia Glycine Decarboxylase p-subunit* (FtGLDp) promoter activity and chloroplast proliferation in the BS of the *pFtGLDp::OsCGA1* transgenic lines**

(**A** and **C**) Schematic of *FtGLDp* promoter GUS constructs. The *GLDp* promoter from *Flaveria trinervia* was fused to *B-glucuronidase* and *OsTubA1::HPT* containing a hygromycin resistance cassette. (**A**) Artificially introduced intron sequences within the GUS gene were from AT1G06220, but not included in (**C**) *pFtGLDp::GUS* minus intron construct. Positions where two intron sequences introduced are indicated as connecting angled lines. (**B** and **D**) Cross-sections of GUS stained leaves from transgenic. (**B**) *FtGLDp::GUS+introns* plants and (**D**) *FtGLDp::GUS-introns* plants. T, nos terminator; L, Left border of transfer DNA; R, right border of transfer DNA. Cross-sectional GUS images are prepared using vibratome sectioning (see online methods). Scale bar, 100 μm. (**E**) T-DNA schematic of p*FtGLDp::OsCGA1* fusion construct. (**F** and **G**) transverse view from confocal images of null (F) and *pFtGLDp::OsCGA1* transgenics (G). Confocal microscopic images are obtained from segregating T2 null (F) and T2 homozygous plants of *pFtGLDp::OsCGA1* #15 events(G). Pseudo-colored grey and red images show cell wall staining (Calcofluor white) and chlorophyll autofluorescence, respectively. Scale bar,10 μm.

**Supplementary Figure 3. Accumulation of photosynthetic enzymes in WT and Transgenic lines**

TEM images showing immunohistochemical detection of (**A** and **B**) Rubisco, (**C** and **D**) Rubisco activase, (**E** and **F**) FBPase and (**G** and **H**) glycine decarboxylase in bundle sheath chloroplasts and mitochondria of the null segregant (A, C, E and G) and *pFtGLDP::OsCGA1* #12 T2 lines (B, D, F and H). Black particles indicate positive immunodetection events. C, chloroplast; M, mitochondria. Bars, 500 nm.

**Supplementary Figure 4. Leaf photosynthetic and biochemical responses**

(**A**) Leaf net CO_2_ assimilation rates (*A*) versus intercellular CO_2_ partial pressure (*C*_i_) determined in the two *pFtGLDp::OsCGA1* transgenic rice lines and their two corresponding null segregants under atmospheric O_2_ partial pressure of 18.4 kPa. Values are mean ± SE (four biological replicates). (**B**) Bar graphs indicating the mean values ± SE (four biological replicates) for (a) Rubisco activity, (b) content of chlorophyll *a*+*b*, (c) specific leaf mass (SLM), and (d) total Nitrogen content expressed per unit leaf surface area in the two transgenic rice lines and the two-corresponding null segregants. In panel (b), dots and error bars represent the mean ratio of chlorophyll *a*/*b* (identified on the secondary Y axis) ± SE for each plant genotype (four biological replicates). For (a), (b) and (c) panels no statistical significance among genotypes was determined based on one-way ANOVA and Tukey *post hoc* test (α= 0.05); in panel (d), the different letters indicate statistically significant differences.

**Supplementary Figure 5.** **Summary of leaf gradient RNA seq in two independent events of *pFtGLDP::OsCGA1***

(**A**) Expression level (TPM) of the *pFtGLDp::OsCGA1* transgene and *OsCGA1* endogenous gene at specific developmental stages along a developing 4th leaf in transgenic (T) and corresponding null (N) lines. The black bars and empty bars represent transgenic *OsCGA1* and endogenous *OsCGA1* expression, respectively. B; Base, -1; -1 cm segment below covered by outer leaf, +4; 4 cm above emergence point, T; tip region. Endogenous *OsCGA1* expression peaked at the base region of the leaf and was maintained at low levels throughout leaf development. The *pFtGLDp*-driven expression of *OsCGA1* was inverse to the native OsCGA1 gene, showing a gradual increase in expression from base to tip, suggesting that *pFtGLDp* activity increases as the leaf matures, consistent with the role of GLD in photorespiration [(Bauwe *et al.*, 2010)](https://paperpile.com/c/PATKsO/GniW). Differential gene expression analysis between transgenic and the null lines was conducted in the +4 and tip region, where transgene expression peaked. Using a relatively non-stringent false discovery rate cut off of 0.1, only 6 and 7 genes in +4 and tip regions, respectively, were identified as DE in two independently transformed lines. (**B**) Summary of differentially expressed genes in +4 and tip segment between transgenic and null (T Vs N). (**C**) Heat map of DE genes enriched in + 4 and tip leaf segments in both *pFtGLDP::OsCGA1* #12 and #15 events independently.

**Supplementary Figure 6.** **Microdissection images of BS strands and M strands and their RNA profiles from *pFtGLDP::OsCGA1* transgenic and nulls*.***

Mesophyll and bundle sheath stands were captured separately on capsure caps from paradermal sections of rice leaves. Higher magnification of captured tissue show little cross-contamination between mesophyll and bundle sheath strands, bioanalyzer traces of RNA extracted from captured tissue shows high quality RNA with clear 18S and 25S rRNA peaks, and more apparent chloroplastic rRNA (CP rRNA) in mesophyll samples compared with bundle sheath strands in both null (#12-2-2) and transgenic plants (#12-4-4).

**Supplementary Figure 7. Pagemen analysis using DE genes in BSS LCM seq.**

Major bin categories identified using the Pageman tool [(Usadel *et al.*, 2006)](https://paperpile.com/c/PATKsO/Gs2c) with a Wilcoxon test (FDR<0.05) transcripts accumulating to significantly higher (red) or lower (blue) level in bundle sheath strands (BSS) of transgenic lines in comparison with null segregants.

**Supplementary Figure 8. Transcriptional activation test on *OsCGA1* *promoter GUS* reporter using dCas9-mediated transactivation in heterologous system *Nicotiana tabaccum.***

(**A**) Schematic of constructs to test individual gRNA efficacy for transactivation of the *pOsCGA1::GUS* reporter. The *pOsR1G1b::DsRED* cassette was used for filler sequence to link the reporter cassette and dCas9-AC cassette. (**B**) Summary of gRNA sequences which were designed to target the OsCGA1 promoter region. gRNA position was indicated as distance from the transcriptional start site. Target sequence was represented with a 20bp gRNA complementing region and PAM motif (Bold). (**C**) GUS staining of tobacco leaf discs inoculated with the dCas9 activation constructs. Two different stages of fully emerged leaves were infiltrated with agrobacterium suspension for each construct. After 4 days, four, 1 cm wide leaf discs were subject to the gus solution for 17 hours. Each of the four-leaf discs shown represents one inoculation for each construct. Scale bar, 2mm.

**Supplementary Figure 9. Tissue specific expression of *pOsCGA1::GUS* reporter by dCas9 mediated activation in *Kitaake* transgenics**

Transverse sections of GUS-stained leaves from dCas9 activation lines with different gRNAs. (**A**) *pFtGLDp::GUS* line*,* pDLGG039 #4*,* was included as a control for the comparison of GUS stained intensities with 17 hrs incubation time. (**B**) dCas9-ADs transgenic, pDLGG047 #4, without gRNAs (**C**) pDLGG048 #10, gRNA2 (**D**) pDLGG048 #14, gRNA3. (**E**) pDLGG049 #1, gRNA4 (**F**) pDLGG054 #4, gRNA4. Transverse sections were prepared on the vibratome after rehydration (see online methods). gRNA species of each construct are shown in the figures. Red arrow indicates the BS cells with cytoplasmic GUS stains encompassed in the 50 μm thick vibratome section. Scale bar, 10 μm.

**Supplementary Figure 10. Chloroplast morphologies in the bundle sheath cells of *dCas9* activation lines**.

(**A-D**) longitudinal and (**E-H**) transverse sections of leaf segments. (**A**) chloroplasts were uniformly distributed in the periphery of the BS of pDLGG047 #4 (no gRNA). (**B**) chloroplasts aggregated preferentially in the central region of the BS in lines carrying pDLGG054 #1 (gRNA1,2,3). (**C**) stromules (yellow arrow) appear more frequently in chloroplasts of pDLGG048 #14 (gRNA2) leaves (**D**) chloroplasts appear to aggregate in the vacuole of BS in pDLGG053 (gRNA 1,2,3). (**E**) major vein of pDLGG047 #4 (no gRNA). (**F**) major vein of pDLGG053 #1. Chloroplasts proliferation is prevalent in multiple vascular parenchyma cells in dCas9 activation lines (white arrowhead) compared to the pDLGG047. (**G**) major vein of pFtGLDp::OsCGA1 #12. Chloroplast aggregation (white arrow) is occasionally observed in some BS cells. (**H**) minor vein image of pDLGG048 #14 (gRNA2). Stromules (yellow arrow) and diffuse chlorophyll autofluorescence signals are shown in the BS cells. Fixed leaf segments were embedded in 7% agarose and sliced on the vibratome. Cyan and red colors are shown as pseudo color of calcofluor staining and chlorophyll autofluorescence, respectively in the confocal image stacks. MX, metaxylem; MS, mestome sheath; BS, bundle sheath; M, mesophyll. Scale bar,10 μm.

|  |  | *Null* | *pFtGLDp::OsCGA1* | | |
| --- | --- | --- | --- | --- | --- |
|  |  | #12-17 | #2-13 | #12-15 | #15-19 |
| Mitochondria planar area/Planar cell area, % | BS | 0.49 ± 0.6^a^ | 0.37 ± 0.2^a^ | 0.36 ± 0.2^a^ | 0.82 ± 0.7^b^ |
| Mitochondria number /Planar cell area, µm^2^×10^-3^ | BS | 28.7 ± 26.9^a^ | 25.4 ± 15.6^a^ | 24.2 ± 15.2^a^ | 44.0 ± 32.6^b^ |
| Peroxisome planar area/Planar cell area, % | BS | 0.13±0.3^a^ | 0.10±0.3^a^ | 0.18±0.4^a^ | 0.27±0.5^a^ |
| Peroxisome number /Planar cell area, µm^-2^ ×10^-3^ | BS | 2.8±5.3^a^ | 3.4±6.4^a^ | 4.1±6.9^a^ | 4.2±1.0^a^ |

**Supplementary Table 1. TEM and quantitative measurements of organelle phenotype in *pFtGLDp::OsCGA1* transgenic**

Quantification of planar area of organelles in bundle sheath cells of WT and transgenic lines. Values are mean ± SD (n = 3 [10 -15 cells/3 individuals]). ^a-d^ values with the same letters represent no significant difference (p > 0.05) by Kruskal Wallis one way analysis of variance followed by a Dunn's test.

*These values are quantified from two biological replicates.

**Supplementary Methods**

**GUS staining procedures for expression analysis**

GUS staining of rice leaf tissue is challenging due to the epidermal wax barrier that prevents permeation of GUS substrates. It was thus necessary to strip epidermal wax cuticles by dipping leaf segments in chloroform then washed immediately 3 times in water. Washed leaf segments were vacuum infiltrated in a GUS substrate solution (0.8 mg/ml 5-bromo-4-chloro-3-indolyl-β-D-glucuronic acid (X-GLUC), 8mM EDTA, 1mM potassium Ferricyanide, 1mM potassium Ferrocyanide, 0.1% Triton X-100, and 0.2% DMSO in 80 mM Sodium phosphate buffer) until leaf tissues sunk in the GUS solution. GUS-stained samples were incubated at 37°C for 2 days (pFtGLDp::GUS) or 17 hrs (dCas9 activation lines). After GUS staining, tissues were cleared in 70% ethanol until dechlorophyllized. To visualize GUS stained tissues were rehydrated with reverse-ethanol series (70% → 50% → 30% →0 % Et-OH) and embedded in the 7% low melting agarose. Thick cross sections, 60~70 μm, were cut on a vibratome and mounted on the slides.

**DNA gel blot and Taqman copy number analysis for transgene characterization**

Genomic DNA was extracted from rice leaves using the standard CTAB extraction method [(Chen and Ronald, 1999)](https://paperpile.com/c/PATKsO/3ddYz). For each plant, 1 ug of genomic DNA was digested with HindIII (pDLGG002) or PstI (pDLGG003) restriction endonuclease. Digested DNA was resolved on a 1% agarose gel and then transferred to Hybond N+ membrane (GE Healthcare,Chicago, USA). After UV-crosslinking, transferred membrane was hybridized with the digoxigenin (DIG)-labelled probes for hygromycin phosphotransferase (HPT) gene or nopaline synthase terminator (Nos T) sequence in DIG Easy Hyb buffer (Roche Diagnostics,[Basel, Switzerland](https://www.google.com/search?rlz=1C1CHBD_enUS831US831&sxsrf=ACYBGNQjigMO_4aLk1B6hXsjudRcY5RkTQ:1568579565767&q=Basel&stick=H4sIAAAAAAAAAONgVuLQz9U3MMw2SXvE6Mgt8PLHPWEpi0lrTl5jNOLiCs7IL3fNK8ksqRRS4WKDsqS4eKTgmjQYpLi44DyeRaysTonFqTkAgDrftlgAAAA)) at 65°C. The DIG Probe was generated by PCR using primers shown in **Table S3** with DIG DNA labelling Mix (Roche Diagnostics). After hybridization, blots were washed (2 x SSC and 0.1% SDS) two times. CDP-star was used for signal detection of DNA bands of interest as manufacturer’s instructions (Roche Diagnostics, Basel, Switzerland). Membranes was stripped off with a 0.2 N NaOH, 1% SDS solution and reprobed with another probe. Transgene copy number by individual probes are shown in the **Table S2**.

Taqman copy number analysis was used for genotyping. Approximately 10 ng of RNA free genomic DNA was used as a template for Taqman PCR analysis with TaqMan Genotyping Master mix (Life Technologies, Carlsbad, USA) using a LightCycler 480 II (Roche Diagnostics). Probe and primer sets for the target gene (HPT and NosT) and reference gene (RFL, LOC_Os04g51000) were synthesized by Integrated DNA Technologies (Coralville,USA) as shown in **Table S3**. A Cp value, ‘Abs Quant/2nd Derivative Max’ was determined using the manufacturer’s software, and a standard ddCt method of analysis was used for each probe set. Known heterozygous single copy T-DNA lines were used as a standard calibrator and the average of 2^-⊿⊿Cp^ is shown as copy number of T-DNA regions in **Table S2**.

**Leaf photosynthetic analysis**

The response curves of leaf net CO_2_ assimilate rates per unit leaf surface area (*A*, *µ*mol CO_2_ m^-2^ s^-1^) to intercellular CO_2_ partial pressure (*C*_i_, Pa) were determined on 30-40 day old plants for each rice genotype (four biological replicates) based on measurements of leaf-atmosphere CO_2_ exchange to atmospheric partial pressure of CO_2_ (*C*_a_). For each plant, the analysis was conducted on the youngest fully developed leaf (usually the third emerged) of two central stems in the canopy. Measurements were taken between 10:00 a.m. and 4:00 p.m. standard time with a LI**-**COR 6400XT portable photosynthesis system (LI**-**COR Biosciences, NE, USA) equipped with a 2 x 3 cm leaf chamber fitted with a 6400**-**02B LED light source. For each plant, two mid-distal leaf blade portions were simultaneously included in the chamber to cover the whole lumen section. Measurements were conducted under atmospheric O_2_ partial pressure of 18.4 kPa, and PPFD was set at 1500 *µ*mol photons m^-2^ s^-1^, leaf temperature at 25 °C, and leaf**-**to**-**air VPD in the range of 1.0**-**1.5 kPa. To determine the *A*/*C*_i_ response curve, leaf chamber *C*_a_ was changed stepwise from current ambient level (~ 36.8 Pa, *i.e.* ~ 400 *µ*mol CO_2_ mol^-1^ air) down to 3.7 and up to 184.2 Pa through total 14 *p*CO_2_ levels. More precisely, the imposed *C*_a_ sequence was 36.8, 27.7, 18.4, 13.8, 9.2, 6.9, 3.7, 9.2, 18.4, 36.8, 55.3, 73.7, 138.2, 184.2 Pa, and the leaf blade portions were kept at each *C*_a_ for a minimum of 120 and maximum of 180 s durations. During this time interval, after programmed matching of the LI-6400XT gas analyzers, the data were automatically recorded.

**Leaf biochemical analysis**

For all rice genotypes (four biological replicates), a leaf extract was made from leaves used for gas exchange measurements. Rubisco activity (*µ*mol CO_2_ m^-2^ s^-1^) was determined according to [(Walker *et al.*, 2013)](https://paperpile.com/c/PATKsO/j4Hn). Chlorophyll *a* and *b* were extracted in 100% ethanol and chlorophyll absorbance was measured at 665 and 649 nm, respectively, on a Graphicord UV-240 Shimadzu spectrophotometer. The data were analyzed as in Porra *et al.* (1989) to determine chlorophyll content (*a*+*b*; chl *a*/*b*, g m^-2^).  Additionally, leaf tissue was dried in an oven at 55 °C for 48 h to calculate the specific leaf dry mass (SLM, g m^-2^). Total leaf Nitrogen content (Total N, g m^-2^) was determined through dry matter combustion in an elemental analyzer (Costech ECS 410, Valencia, CA, USA), followed by N_2_ gas admission into a stable isotope ratio mass spectrometer (GV Instrument Isoprime, Manchester, UK).

**Statistical analysis**

Statistical analysis was conducted for Rubisco activity, chl *a* + *b*, chl *a*/*b*, SLM, total N, and *A* determined at each *C*_i_ and was performed by one-way ANOVA followed by Tukey *post hoc* test for pairwise comparisons of means (α= 0.05) using OriginPro 2018b software (OriginLab Corp., MA, USA).

**Leaf gradient RNA seq generation**

For RNA seq analysis, pFtGLDp::OsCGA1 #12 and #15 lines were back-crossed to the *Kitaake* wild-type. Fixed homozygotes and their null segregants from BC1F2 populations were used for the RNA seq analysis. The 1cm segments of base, below ligule, 4cm above ligule and tip regions were harvested from the 4th developing leaves of #12, #15 BCF2 lines at midday. 3’ mRNA -seq libraries were generated using a QuantSeq 3’ mRNA-Seq Library prep kit (Lexogen, Vienna, Austria). 1ug total RNA and 13 PCR cycles were used for the generation of individual libraries according to manufacturer’s recommendations. Pooled libraries were sequenced using 100-bp single end reads on an Illumina HiSeq 4000 system.

**Tobacco leaf infiltration for the transient dCas9 activation**

Our tobacco leaf infiltration method was adapted from [(Kapila *et al.*, 1997)](https://paperpile.com/c/PATKsO/Hg3l). Constructs from pDLGG027~pDLGG032 were transformed into *Agrobacterium* strain *AGL1*. After validation of *Agrobacterium* transformation, strains were inoculated in 2 mL LB (50 μg/ml of kanamycin) at 28 ℃ with vigorous shaking overnight (250 rpm). Approximately 17 hrs after inoculation, cells were harvested by centrifugation at 4000 rpm for 5 minutes. Agro pellets were resuspended with MES buffer (10mM MES-KOH, 10 mM MgCl2, pH 5.6) with 40 μg/ml acetosyringone up to 0.2 of OD_600_. The cells were incubated at 28 ℃ for 2 hours to induce the virulence. The abaxial side of the leaf was abraded with the sharp end of the 18g needle. A syringe was then used to gently push the agro suspension through the opening by placing a finger on the opposite side of the leaf and gently infiltrating the leaf using the hypodermic syringe. The first two fully emerged leaves from the top of the plant were subjected to infiltration. Plants were allowed to recover at room temperature for 4~5 days before GUS staining.

**Supplementary References**

[Bauwe, H., Hagemann, M., and Fernie, A.R. (2010) *Photorespiration: players, partners and origin*. *Trends Plant Sci.*, **15**, 330–336.](http://paperpile.com/b/PATKsO/GniW)

[Chen, D.-H. and Ronald, P.C. (1999) *A Rapid DNA Minipreparation Method Suitable for AFLP and Other PCR Applications*. *Plant Mol. Biol. Rep.*, **17**, 53–57.](http://paperpile.com/b/PATKsO/3ddYz)

[Jeon, J.S., Lee, S., Jung, K.H., Jun, S.H., Kim, C., and An, G. (2000) *Tissue-preferential expression of a rice alpha-tubulin gene, OsTubA1, mediated by the first intron*. *Plant Physiol.*, **123**, 1005–1014.](http://paperpile.com/b/PATKsO/xU9j)

[Kapila, J., De Rycke, R., Van Montagu, M., and Angenon, G. (1997) *An Agrobacterium-mediated transient gene expression system for intact leaves*. *Plant Sci.*, **122**, 101–108.](http://paperpile.com/b/PATKsO/Hg3l)

[Porra, R.J., Thompson, W.A., and Kriedemann, P.E. (1989) *Determination of accurate extinction coefficients and simultaneous equations for assaying chlorophylls a and b extracted with four different solvents: verification of the concentration of chlorophyll standards by atomic absorption spectroscopy*. *Biochimica et Biophysica Acta (BBA) - Bioenergetics*, **975**, 384–394.](http://paperpile.com/b/PATKsO/T3oD)

[Usadel, B., Nagel, A., Steinhauser, D., Gibon, Y., Bläsing, O.E., Redestig, H., et al. (2006) *PageMan: an interactive ontology tool to generate, display, and annotate overview graphs for profiling experiments*. *BMC Bioinformatics*, **7**, 535.](http://paperpile.com/b/PATKsO/Gs2c)

[Walker, B., Ariza, L.S., Kaines, S., Badger, M.R., and Cousins, A.B. (2013) *Temperature response of in vivo Rubisco kinetics and mesophyll conductance in Arabidopsis thaliana: comparisons to Nicotiana tabacum*. *Plant Cell Environ.*, **36**, 2108–2119.](http://paperpile.com/b/PATKsO/j4Hn)
